# Facile Fabrication of Double-Shell PA-MEL@PU@CFO Microcapsules and Their Fire Suppression Performance in Confined Spaces

**DOI:** 10.3390/ma19194135

**Published:** 2026-09-28

**Authors:** Zhe Wen, Zihan Wang, Lingfeng Xiong, Hongxiang Ou, Fang Zhu, Honglai Xue, Yingchu Wang

**Affiliations:** 1 School of Petrochemical Engineering, Changzhou University, Changzhou 213164, China; wenzhe@cczu.edu.cn; 2 Jiangsu Province Key Laboratory of Fine Petrochemical Engineering, Changzhou University, Changzhou 213164, China; 3 School of Safety Science and Engineering, Changzhou University, Changzhou 213164, China; 4 State Key Laboratory of Fire Science, University of Science and Technology of China, Hefei 230026, China

**Keywords:** microcapsules, perfluorohexanone, phytic acid-melamine salt, fire suppression performance, confined-space fires, thermal runaway of lithium-ion batteries

## Abstract

Novel double-shell PA-MEL@PU@CFO microcapsules were successfully prepared by further coating the as-prepared PU@CFO microcapsules with the outer shell of phytic acid-melamine salt (PA-MEL). The fire suppression performance was then investigated in a simulated confined space experimental device. Effects of the emulsification conditions on the morphology of microcapsules were investigated in detail. Systematic characterization results demonstrated that the double-shell structure was successfully constructed. PA-MEL@PU@CFO microcapsules fabricated under the optimal emulsification conditions exhibited regular spherical morphology with rough flocculent surface and uniform particle size. The introduction of the outer PA-MEL shell raises the release temperature of core material, thereby improving the low-temperature storage stability of the microcapsules and facilitating their storage and transportation. PA-MEL@PU@CFO microcapsules exhibited favorable storage stability overall, with a relatively high core content of 53.18%. PA-MEL@PU@CFO microcapsules demonstrated excellent flame retardant and fire-extinguishing performance for the fires in confined spaces at the incipient fire stage, reducing the maximum flame temperature by 238.6 °C, shortening the extinguishing time by 53 s, and effectively inhibiting flame re-ignition. PA-MEL@PU@CFO microcapsules achieved efficient fire suppression by a synergistic effect of physical cooling, chemical suppression and phosphorus–nitrogen synergistic flame retardancy. PA-MEL@PU@CFO microcapsules hold great practical application potential in practical application such as anti-flame coatings and lithium-ion batteries.

## 1. Introduction

Lithium-ion batteries have been widely adopted in electric vehicles, energy storage systems and consumer electronics for the high energy density [1,2,3]. Nevertheless, lithium-ion batteries suffer from high risk of thermal runaway, which easily triggers confined-space fires with rapid flame spread [4,5]. In confined battery systems, conventional fire extinguishing agents fail to penetrate deep into the fire source, resulting in delayed fire suppression and frequent re-ignition [6,7]. Development of timely, rapid, and efficient fire-extinguishing technologies remains a critical research hotspot, which is of great practical significance for ensuring the safe production and healthy development of the lithium-ion battery industry.

Microcapsule fire extinguishing agents are prepared via microencapsulation of fire extinguishing agents within polymer shells, where fire extinguishing agents are taken as the core materials to construct the microscale core–shell structured capsules [8,9]. The primary purpose of microencapsulation technology is to isolate the core materials from the external environment, thus achieving the protection, preservation and controlled release of active core materials [10]. Once the ambient temperature reaches the designed response temperature, the shell layers will be thermally ruptured and rapidly release the internal fire extinguishing agents to extinguish fires [11]. Microcapsule fire extinguishing agents characterized by active defense properties can effectively restrain the thermal runaway of lithium-ion batteries through various ways, such as embedding inside lithium-ion battery, coating on battery casings, and filling the gaps between battery modules [12,13,14]. Perfluorohexanone (CFO) serves as an eco-friendly clean liquid extinguishing agent alternative to halons, characterized by high fire-extinguishing efficiency, low toxicity, zero ozone depletion potential, no residue after volatilization and a moderate boiling point [15]. Encapsulated CFO has been widely investigated for confined energy storage systems such as power stations, battery packs, and precision electronics [16,17]. Conventional preparation methods of CFO-based microcapsules mainly include interfacial polymerization [18], in situ polymerization [19] and complex coacervation [20]. Xiao et al. prepared the fire extinguishing microcapsules using CFO as the core materials and polyurea as the wall materials, and further fabricated a bifunctional fire-extinguishing microcapsule-coated alumina/silica aerogel sheet [21]. The fire extinguishing microcapsules coated aerogel sheet could release fire-extinguishing agent in the early stage of thermal runaway by rapid heat absorption and suppressed further thermal runaway propagation by prohibiting chemical chain reaction and heat transfer. The thermal runaway propagation of the battery module in a confined space was delayed from 430 s to 2907 s. Meng et al. prepared the fire extinguishing microcapsules using CFO as the core materials and urea-formaldehyde resin as the shell material, and further synthesized a novel Fire-extinguishing Polymer Composite by the in situ polymerization of microcapsules and epoxy resin materials [22]. The encapsulation efficiency of the obtained microcapsules reached 70% and the response temperature was approximately 120 °C. Laboratory-scale fire extinguishing performance tests demonstrated that the Fire-extinguishing Polymer Composite could rapidly respond to release CFO and effectively inhibited thermal runaway in lithium-ion batteries. Guo et al. also prepared the fire extinguishing microcapsules using CFO as the core materials and urea-formaldehyde resin as the shell material, and directly coated the CFO-based microcapsules on the outer surface of the lithium-ion battery [23]. Results showed that the introduction of microcapsules could automatically detect and respond at the early stage of battery thermal runaway. The formation and propagation of thermal runaway were effectively inhibited with delayed thermal runaway propagation of 250 s. As concluded, CFO-based microcapsules exhibit remarkable suppression performance for the thermal runaway of lithium-ion batteries.

In the previous work, we have reported the preparation of single-shell PU@CFO microcapsules via interfacial polymerization using CFO as the core materials and polyurea (PU) as the wall materials, and the fabrication of double-shell MF@PU@CFO microcapsules by further coating PU@CFO microcapsules with flame-retardant melamine formaldehyde (MF) resin [24]. The double-shell MF@PU@CFO microcapsules could effectively overcome the insufficient anti-reignition performance of single-shell microcapsules by the introduction of the outer shell with perfect flame-retardant performance. MF@PU@CFO microcapsules demonstrated excellent fire-extinguishing and flame-retardant performance, reducing the maximum flame temperature by 255.6 °C, and shortening the self-extinguishing time by 36 s. Given that MF resin is synthesized by stepwise polycondensation crosslinking reactions of melamine (MEL) and formaldehyde, residual formaldehyde may remain in the materials followed by gradual sustained release in the subsequent service process, posing potential hazards to human health and ecological environment. Phytic acid (PA) is a natural polyorganic phosphoric acid widely distributed in cereals and oil crops, which serves as one of the most widely utilized bio-derived flame retardants [25]. PA can replace formaldehyde as a crosslinking component and react with MEL to form water-insoluble flame retardant of phytic acid–melamine salt (PA-MEL) [26]. The reaction between PA and MEL is essentially an ionic crosslinking reaction based on acid–base neutralization, which is also assigned to a self-assembly reaction. Since no formaldehyde is involved throughout synthesis and application, PA-MEL exhibits superior environmental benignity, which can fundamentally eliminate the inherent drawbacks of residual formaldehyde and its subsequent slow release in MF resins.

In this study, PA-MEL was adopted to coat the as-prepared PU@CFO microcapsules to fabricate double-shell PA-MEL@PU@CFO microcapsules. The effects of the emulsification conditions on the morphology and core content of microcapsules were studied systematically. A series of systematic characterizations were performed on PA-MEL@PU@CFO microcapsules to investigate their surface morphology and thermal degradation properties. Core content and storage stability of microcapsules were also measured. Finally, fire suppression performance tests of microcapsules were carried out in a simulated confined space experimental device using n-heptane as fuel.

## 2. Experimental

### 2.1. Materials

All chemicals were commercially available and used without further purification. Perfluorohexanone (CFO, ≥98% purity), methyl nonafluorobutyl ether (MNFB, ≥98% purity), octylphenol polyoxyethylene (10) ether (OP-10, Analytical Reagent Grade), formaldehyde (37.0–40.0%, Analytical Reagent Grade) and phytic acid (PA, Bio-Active Grade) were purchased from Shanghai Titan Technology Co., Ltd. (Shanghai, China). Isophorone diisocyanate (IPDI, 99% purity) and diethylenetriamine (DETA, ≥99% purity) were purchased from Shanghai McLean Biochemical Technology Co., Ltd. (Shanghai, China). Melamine (MEL, 99% purity), glacial acetic acid (HAc, Analytical Reagent Grade) and sodium hydroxide (NaOH, Analytical Reagent Grade) were purchased from Shanghai Lingfeng Chemical Reagent Co., Ltd. (Shanghai, China). Deionized water was prepared using a Master-S15 ultrapure water purification system (Hitech Instruments Co., Ltd., Shanghai, China).

### 2.2. Preparation of Double-Shell PA-MEL@PU@CFO Microcapsules

The single-shell PU@CFO microcapsules were prepared by interfacial polymerization method. The preparation process of PU@CFO was shown in Figure 1a and the detailed preparation procedure was presented in the Supplementary Materials. The double-shell PA-MEL@PU@CFO microcapsules were fabricated by further coating the as-prepared PU@CFO microcapsules with PA-MEL and the coating process is exhibited in Figure 1b. Specifically, 0.5 g of MEL was dispersed in 50 mL of deionized water in a round-bottom flask under mechanical stirring at 80 °C for 10 min to obtain the uniformly dispersed MEL solution. A total of 0.62 g of PA was dispersed in 10 mL of deionized water under mechanical stirring at 80 °C for 30 min to obtain the uniformly dispersed PA solution. PA solution was added dropwise into the MEL solution and the PA/MEL mixed solution was continuously stirred at 80 °C for 30 min. A total of 3 g of PU@CFO microcapsules was dispersed in 150 mL of deionized water to obtain the well-dispersed and stable PU@CFO suspension after first ultra-sonication for 30 min and subsequent mechanical stirring for 30 min. The PA/MEL mixed solution was added dropwise into the PU@CFO suspension and then mechanically stirred at 80 °C for 2 h. PA-MEL@PU@CFO microcapsules were finally obtained after filtration, washing and freeze-drying.

### 2.3. Characterization of Microcapsules

The surface morphologies and elemental composition of PU@CFO and PA-MEL@PU@CFO microcapsules were measured with a SUPRA-55 field-emission scanning electron microscope (FE-SEM) (Zeiss, Oberkochen, Germany) coupled with energy dispersive spectroscopy (EDS) at an accelerating voltage of 10 kV after gold sputtering. Thermogravimetric (TG) measurements were conducted on a TGA 209 F1 Libra thermogravimetric analyzer (Netzsch, Selb, Germany). The measurements were carried out from 50 to 800 °C in nitrogen flow of 40 mL/min and the heating rate was 10 °C/min. FT-IR spectra of PU@CFO and PA-MEL@PU@CFO microcapsules were recorded on a TENDOR 27 Fourier transform infrared spectrometer (Bruker, Bremen, Germany) using the KBr pellet method. The scanning range was set from 400 to 4000 cm^−1^ and the spectral resolution was 4000 cm^−1^.

### 2.4. Fire Suppression Performance Test

As shown in Figure 2, fire suppression performance tests of microcapsules were carried out in a semi-enclosed chamber with dimensions of 35 cm × 35 cm × 35 cm. The reaction chamber was relatively sealed with a small hole preserved on the side wall and 9 small holes arranged on the top wall to prevent flame self-extinguishing caused by oxygen deficiency. The combustion test was conducted in a crucible using n-heptane as fuel. Typically, 2 mL of n-heptane was placed in the combustion crucible and subsequently ignited before the test. A total of 2 g of microcapsules were placed on a stainless steel mesh, which was located 10 cm above the fuel surface. A Type K thermocouple (Shanghai Automation Instrument Co., Ltd., Shanghai, China) with measuring range from −73 °C to 705 °C and accuracy of ±1.5 °C was installed 8 cm above the fire to measure the temperature variation of fire scene. The temperature data were recorded by a K-type thermocouple thermometer (Shanghai Automation Instrument Co., Ltd., Shanghai, China) with accuracy of ±0.2% and sampling rate of 1 time/second, which were then transferred to the computer via the temperature data recorder (SUPCON Technologies Co., Ltd., Hangzhou, China).

## 3. Results and Discussion

### 3.1. Characterization of PA-MEL@PU@CFO Microcapsules

During the interfacial polymerization process, emulsification conditions have significant influences on the morphology of the final single-shell microcapsules [27,28,29]. In this study, the effects of emulsification conditions including emulsification rotation speed, emulsification time, IPDI/DETA monomer ratio and CFO loading amount on the morphology of the obtained PU@CFO microcapsules were investigated in detail. The results were presented in the Supplementary Materials, denoted as Figures S1–S8. In summary, the optimal emulsification conditions for preparing PU@CFO microcapsules are as follows: of 3000 rpm, of 10 min, IPDI/DETA of 1:2.5, and CFO of 4 mL. The PU@CFO microcapsules fabricated under the optimal emulsification conditions exhibit regular morphology and uniform particle size. PA-MEL@PU@CFO microcapsules in this study were prepared by further coating the single-shell PU@CFO microcapsules prepared under the optimal emulsification conditions with PA-MEL. Systematic characterizations including SEM, FT-IR, and TG/DTG analyses were carried out to clarify the morphology and structure of the double-shell PA-MEL@PU@CFO microcapsules.

#### 3.1.1. SEM Analysis of PA-MEL@PU@CFO Microcapsules

Integrity and compactness of the core–shell structure are crucial for the thermal stability of microcapsules. SEM serves as an essential characterization method for assessing the microscopic morphology and structural integrity of microcapsules. Moreover, the compactness and defects of the shell can be observed clearly and precisely by SEM.

The SEM images of single-shell PU@CFO and double-shell PA-MEL@PU@CFO microcapsules were exhibited in Figure 3. As shown in Figure 3a, PU@CFO exhibited excellent microscopic morphology, characterized by a regular spherical morphology with smooth surface and uniform particle size of approximately 1.8 μm. No obvious agglomeration was observed. During the interfacial polymerization of IPDI and DETA, the two primary amino groups and one secondary amino group (-NH_2_ and -NH, acting as nucleophiles) in DETA molecular react with the isocyanate groups (-NCO, acting as electrophile) at both ends of IPDI molecular via a nucleophilic addition reaction with hydrogen transfer [30]. Rapid cross-linking reaction takes place at the oil/water interface to construct a three-dimensional network structure. This reaction produces polyurea polymers containing urea linkages (-NH-CO-NH-), thus achieving the effective encapsulation of the core material CFO by the dense polyurea shell. As exhibited in Figure 3b, PA-MEL@PU@CFO was also characterized by a regular spherical morphology with uniform particle size of approximately 2.0 μm. Different from the smooth surface of PU@CFO, the surface of PA-MEL@PU@CFO was covered with a layer of flocculent material. The flocculent material was the PA-MEL salt synthesized by PA and MEL. According to the reaction mechanism, negatively charged phosphate groups are released when PA dissociated in water. These groups can combine with the positively charged amino groups in MEL molecular to form water-insoluble PA-MEL. The water-insoluble PA-MEL then precipitates from water and aggregates on the surface of PU@CFO microcapsules to form the double-shell PA-MEL@PU@CFO microcapsules.

To further determine the composition of the PA-MEL@PU@CFO microcapsules, EDS elemental mappings including carbon (C), oxygen (O), phosphorus (N), and fluorine (F) were analyzed and shown in Figure 3c. EDS results revealed the presence of characteristic elements of core and shell materials in the spherical core–shell microcapsules. C and O, which are characteristic elements of the PU shell, were densely distributed around the entire spherical surface of the microcapsules, forming a continuous and integral shell layer. The distribution areas of C and O are consistent with the outline of spherical microcapsules, indicating the complete encapsulation of the core material by the PU shell. As the characteristic element of the core material CFO, F was relatively concentrated in the central area of the sphere, confirming the intact storage of the core material in the microcapsules. The content of F elements increased obviously at the central region of the sphere, which clearly distinguished the core region from the shell layer. As the characteristic element of PA-MEL, P was homogeneously dispersed around the entire spherical surface of the microcapsules rather than agglomerated. The distribution area of P highly overlapped with the distribution areas of C and O, and fully covered the distribution area of F. It demonstrated that PA-MEL was successfully and densely adhered to the surface of PU@CFO microcapsules rather than self-agglomeration, achieving double-shell encapsulation of microcapsules. It can be seen from the EDS results that the double-shell structure of PA-MEL@PU@CFO microcapsules are successfully constructed.

#### 3.1.2. TG/DTG Analysis of PA-MEL@PU@CFO Microcapsules

The TG and DTG results of PU@CFO and PA-MEL@PU@CFO microcapsules were shown in Figure 4. PU@CFO and PA-MEL@PU@CFO microcapsules exhibited relatively similar thermal decomposition characteristics seen from the overall TG curves, but significant differences in the DTG curves. This phenomenon can be attributed to the decomposition process being primarily governed by the volatilization of core materials and the decomposition of wall materials. As for the double-shell PA-MEL@PU@CFO microcapsules, the thermal decomposition of outer shell PA-MEL would affect that of the inner core–shell structure of PU@CFO, thereby resulting in differences in the weight loss rate.

The weight loss of single-shell PU@CFO and double-shell PA-MEL@PU@CFO microcapsules can be roughly divided into three distinct stages. The first stage within the temperature range of 30–200 °C is mainly corresponding to the evaporation of water. At this thermal stage, single-shell PU@CFO microcapsules exhibited a mass loss of 11%, while merely 5% for double-shell PA-MEL@PU@CFO microcapsules. The loss rate of PA-MEL@PU@CFO was much lower than that of PU@CFO, primarily due to the reduced proportion of free water in the microcapsules caused by the introduction of the second shell of PA-MEL. The CFO core materials were not released during this thermal stage, although the temperature of which was much higher than its standard boiling point, confirming that CFO was effectively encapsulated within the single-shell and double-shell materials.

The single-shell PU@CFO and double-shell PA-MEL@PU@CFO microcapsules exhibited different mass-loss behaviors during the second stage within the temperature range of 200–300 °C. A mass loss of approximately 35% was observed in the TG curve of PU@CFO, while much higher of 64% for that of PA-MEL@PU@CFO during this thermal stage. As for the single-shell PU@CFO microcapsules, the weight loss at around 220 °C is assigned to the release of the encapsulated CFO. In contrast to the single-shell microcapsules, two distinct DTG peaks can be observed for the double-shell PA-MEL@PU@CFO microcapsules in the 200–300 °C temperature range, corresponding to CFO release at around 250 °C and thermal decomposition of the outer PA-MEL shell at around 290 °C, respectively. As the temperature increased, the microcapsule shell would rupture under the excessive internal phase-transition pressure generated by the rapid vaporization of CFO, leading to subsequently rapid release of the encapsulated CFO. Compared with single-shell PU@CFO microcapsules, the temperature at the maximum mass loss rate originating from core leakage was delayed by 30 °C for double-shell PA-MEL@PU@CFO microcapsules. It was indicated that the introduction of the outer PA-MEL shell raises the release temperature of core material, thereby improving the low-temperature storage stability of the microcapsules and facilitating their storage and transportation. When the PA-MEL@PU@CFO microcapsules were heated above 260 °C, some unstable groups such as amino and hydroxyl groups in the outer shell of PA-MEL would proceed thermal decomposition, leading to the second characteristic mass loss peak at around 290 °C. Since the thermal decomposition of PA-MEL also contributes to the weight loss, the double-shell PA-MEL@PU@CFO microcapsules exhibit a significantly larger weight loss than that of the single-shell PU@CFO microcapsules.

The third stage within the temperature range of 300–400 °C is attributed to the thermal degradation of the PU shell. At around 320 °C, the material PU began to decompose and resulted in the weight loss. In contrast with the intense and sharp PU decomposition peak of PU@CFO, the corresponding peak of PA–MEL@PU@CFO is significantly weakened and broadened. It might be attributed to the fact that the phosphorus-containing decomposition products of PA-MEL could catalyze the earlier and more gradual decomposition of PU into stable char, rather than the rapid and concentrated thermal degradation at a single temperature. Moreover, the inner PU shell would be protected to a certain extent by the inert gases, water vapor and residue generated from the thermal decomposition of the outer PA-MEL shell. As a result, the decomposition rate of the inner PU shell is significantly decreased and the final residual amount is slightly increased. When temperature reached above 400 °C, mass of both PU@CFO and PA-MEL@PU@CFO remained essentially unchanged, indicating the complete decomposition of the microcapsules.

#### 3.1.3. FT-IR Analysis of PA-MEL@PU@CFO Microcapsules

FT-IR spectra of PU@CFO microcapsules, PA-MEL@PU@CFO microcapsules and core-free pure PU shell are displayed in Figure 5. Overall, the absorption peaks at 1629 cm^−1^ and 3353 cm^−1^ are assigned to the stretching vibrations of C=O and N-H bonds in polyurea structure, respectively. The absorption peak at 1539 cm^−1^ corresponds to the bending vibration of N-H bonds. According to the reaction mechanism of polyurea formation, it can be confirmed that polyurea structure is successfully synthesized in both PU@CFO and PA-MEL@PU@CFO microcapsules. No obvious absorption bands attributed to the -NCO groups in IPDI within the range of 2000–2300 cm^−1^ can be observed, indicating that -NCO groups have been completely consumed and no residual IPDI remains in the samples. Furthermore, the absorption peaks at around 1020 cm^−1^ ascribed to stretching vibration of the -CF bonds in CFO can be observed in the FT-IR spectra of PU@CFO and PA-MEL@PU@CFO microcapsules, while they are not present in that of PU shell. The results demonstrated that CFO was encapsulated within microcapsules. In addition, PA-MEL@PU@CFO microcapsules show two additional absorption peaks relative to PU@CFO microcapsules. The absorption peak at around 3140 cm^−1^ corresponds to the stretching vibration of N-H bonds originated from -NH_3_^+^ in PA-MEL. The band at 1572 cm^−1^ is assigned to the stretching vibration of the C=N bond derived from MEL. Combined with SEM and TG results, it can be included that PA-MEL has been successfully coated on the outer surface of single-shell PU@CFO microcapsules, resulting in double-shell PA-MEL@PU@CFO microcapsules.

#### 3.1.4. Core Content of PA-MEL@PU@CFO Microcapsules

Core content is one of the core indicators for evaluating the preparation process and product quality of microcapsules. It directly affects the effective utilization rate of core materials and the application performance of the final microcapsules. Generally, core content determines the actual loading amount of core materials, thus directly affecting the quantity of encapsulated fire extinguishing agent released per unit mass of microcapsules and thereby dominating its fire suppression performance. High core content can greatly reduce the loss of core materials and improve the utilization efficiency of wall materials, which is of great significance for the large-scale industrial production. In addition, core content can indirectly reflect the long-term storage stability of microcapsules and the release characteristics of core materials under thermal conditions.

In this study, 1.0015 g of PA-MEL@PU@CFO microcapsules was firstly ground into powder to rupture the shell structure and then transferred into a vacuum drying oven. The powder was heated at 120 °C for 12 h to achieve the complete release of the core materials. To confirm the complete removal of the core material, additional experiments with extended drying time of 24 h and 36 h were carried out. Results showed that no further weight loss occurred upon prolonged drying. A total of 0.4689 g of residual sample was finally obtained after drying at 120 °C for 12 h. FT-IR analysis of the residual sample (Figure S9) showed that no characteristic stretching vibration band of -CF attributable to CFO at around 1020 cm^−1^ could be observed, further confirming the complete removal of the core material. According to the corresponding calculation formula, the core content of PA-MEL@PU@CFO microcapsules was determined as 53.18%. Compared with our previously reported single-shell PU@CFO microcapsules with a core content of 42.5% [24], the double-shell PA-MEL@PU@CFO microcapsules in this study exhibited a significantly improved core content. It was confirmed that the outer shell of PA-MEL could effectively enhance the compactness of the microcapsule shell and restrain the loss of core materials during preparation, thus markedly enhancing the core content of double-shell microcapsules. In addition, although the core content of the double-shell PA-MEL@PU@CFO microcapsules in this work is higher than that of most single-shell CFO microcapsules in previous reports, further optimization of the fabrication parameters holds great promise to achieve a higher core content.

#### 3.1.5. Storage Stability of PA-MEL@PU@CFO Microcapsules

The storage stability of microcapsules was evaluated via a 30-day storage test under ambient storage conditions. The weight change of microcapsules during the 30-day storage test and SEM image of aged microcapsules were demonstrated in Figure 6. PA-MEL@PU@CFO microcapsules exhibited overall favorable storage stability, as shown in Figure 6a. Compared with the freshly prepared microcapsules, the weight of aged microcapsules decreased by 12% after the storage test. The 12% mass loss might be ascribed to two coupled mechanisms. On the one hand, the high saturated vapor pressure of the volatile core material (i.e., CFO with boiling point of 49 °C) under room temperature drives intrinsic permeation of CFO molecules through the intact shell via a solution-diffusion process [31], and the internal vapor pressure continuously imposes a positive pressure on the shell. PA-MEL@PU composite shell fabricated via interfacial polymerization cannot achieve a fully dense structure without pores. Although no obvious pore defects can be observed from SEM images, numerous tiny cracks and diffusion channels are retained inside the composite shell, leading to continuous leakage of core vapor during long-term storage. Tiny gaps exist at the interface between the inner PU shell and outer PA-MEL shell, serving as preferential diffusion channels for core material leakage. Combined, gaseous CFO tends to diffuse outward through inner shell of PU and outer shell of PA-MEL, resulting in mass loss of microcapsules. On the other hand, the outer PA-MEL shell, an acid–base salt network stabilized by hydrogen bonds, is inherently sensitive to ambient humidity [32]. The hydrophilic phosphate groups from phytic acid absorb free water in air, leading to local swelling and weakening the ionic and hydrogen-bonded cross-links in acid–base salt network [33]. The phosphate ester linkages also undergo slow hydrolysis under humid conditions, rendering the rigid outer shell progressively brittle. As shown in Figure 6b, slight cracks form on the shell of the aged microcapsules under the sustained internal vapor pressure, providing rapid escape pathways for core material and thereby accelerating its leakage. Future work should focus on the hydrophobic modification of the outer shell, enhancement of the shell cross-linking density and hydrolysis resistance, and optimization of packaging conditions. In summary, the microcapsules exhibit limited mass loss during the storage stability test. The shell of aged microcapsules basically remains intact without obvious collapse or other defects. The combined results indicate that the double-shell PA-MEL@PU@CFO microcapsules in this work exhibit adequate storage stability under ambient conditions within the tested period and possess potential for fire-suppression applications after further improvement of the storage stability.

### 3.2. Fire Suppression Performance of PA-MEL@PU@CFO Microcapsules

To further evaluate the fire suppression performance of PA-MEL@PU@CFO microcapsules, two control group experiments were carried out in a simulated confined space experimental device using n-heptane as fuel. One group served as the control group, in which the self-extinguishing process of n-heptane without fire extinguishing agent was recorded. The other group was the experimental group, where the flame extinction behavior of n-heptane with the addition of PA-MEL@PU@CFO microcapsules was tested. The corresponding fire suppression performance test results are shown in Figure 7 and Table 1.

The results of the control group showed that the flame temperature presented typical wavy fluctuations during the self-extinguishing process of n-heptane. In the initial stage, n-heptane undergoes thermal decomposition, leading to the rapid increase in the concentration of combustible gases generated by decomposition. Sufficient oxygen is available at this stage, which significantly accelerates the combustion rate. Therefore, the initial stage for self-extinguishing of n-heptane is dominated by heat release, resulting in a rapid rise of flame temperature to the maximum temperature of 792.2 °C at 22 s with an average heating rate of 36.0 °C/s. In the second stage, the consumption rate of combustible gas gradually exceeds its generation rate as the combustion process proceeds, and the oxygen concentration within the semi-enclosed space begins to decrease due to rapid consumption. As a result, the combustion rate decreased and the flame temperature dropped to the range of 300–500 °C at combustion times of 40–100 s. An obvious secondary temperature rise occurred in the final combustion stage. This phenomenon is attributed to the promoted vaporization and combustion intensity driven by the thermal feedback from high-temperature gas accumulated in the upper layer to fuel surface, as well as the subsequent supplementation of oxygen. The flame was completely extinguished at 159 s once n-heptane was fully consumed.

The results of the experimental group showed distinctly different temperature evolution behaviors compared with that of the control group. After the addition of the single-shell PU@CFO microcapsules, the maximum flame temperature was reduced to 621.7 °C at 48 s and the extinguishing time was shortened to 134 s. Although PU@CFO exhibited favorable fire-extinguishing performance, a slight temperature rise was observed in the final stage of combustion. This indicated that the single-shell microcapsules possessed weaker re-ignition resistance than the double-shell microcapsules. After the addition of PA-MEL@PU@CFO microcapsules, the flame temperature raised to 551.3 °C at 15 s with an average heating rate comparable to that of the control group in the initial stage. Afterwards, no obvious temperature rise of the flame was observed and the maximum temperature of 553.6 °C was achieved at 43 s with a significantly decreased average heating rate of 12.9 °C/s. The flame temperature then presented a sustained monotonic downward trend. No re-ignition phenomenon occurred during the process where the flame gradually decayed until it was completely extinguished at 106 s. As concluded, PA-MEL@PU@CFO microcapsules exhibited excellent fire-extinguishing and flame-retardant performance, reducing the maximum flame temperature by 238.6 °C, shortening the extinguishing time by 53 s, and effectively inhibiting flame re-ignition. Compared with the previously reported double-shell MF@PU@CFO microcapsules [24], PA-MEL@PU@CFO microcapsules exhibited superior fire suppression performance, shortening the extinguishing time by an additional 17 s. It was indicated that the outer PA-MEL shell provided better flame-retardant efficiency than the conventional MF shell under the present experimental conditions in this study.

In summary, PA-MEL@PU@CFO microcapsules effectively suppressed the spread of fire in the early stage and prevent severe secondary temperature rise (re-ignition), corresponding to the initial and second stages in the control experiment. PA-MEL@PU@CFO microcapsules can rapidly respond to abnormal ambient temperature, undergo thermal decomposition and shell rupture, and release the encapsulated CFO within an extremely short period of time. The combustion process can be effectively suppressed by a synergistic effect of physical cooling, chemical suppression and phosphorus–nitrogen synergistic flame retardancy of the double-shell PA-MEL@PU@CFO microcapsules.

### 3.3. Fire Suppression Mechanism of PA-MEL@PU@CFO Microcapsules

Based on the above experimental results in this work (i.e., the reduced maximum flame temperature and shortened extinguishing time), together with the previously reported fire-extinguishing mechanism of CFO [34,35] and the phosphorus–nitrogen (P-N) synergistic flame-retardant mechanism of PA-MEL [36,37], a plausible fire suppression mechanism is proposed and schematically illustrated in Figure 8. It is reasonable to infer that PA-MEL@PU@CFO microcapsules could achieve efficient fire suppression via a multi-coupled synergistic mechanism integrating physical cooling, chemical inhibition, and phosphorus–nitrogen synergistic flame retardancy. To begin with, PA-MEL@PU@CFO microcapsules will rupture at abnormal ambient temperature and enable the rapid release of the encapsulated CFO. CFO will vaporize rapidly once released. The vaporization of CFO absorbs substantial latent heat in the flame zone and reduces the flame temperature, thus achieving effective physical cooling. The physical cooling mechanism of PA-MEL@PU@CFO is directly supported by the present experimental results, as indicated by the reduced flame maximum temperature observed during the fire suppression tests. Meanwhile, we propose that chemical inhibition further promotes the flame quenching. Thermal decomposition of CFO may produce fluorine-containing radicals such as •CF_3_ and •F [38]. The fluorine-containing radicals can terminate the combustion chain reaction by effectively scavenging the active free radicals mainly including •H and •OH in flame. Moreover, by analogy with the gas-phase mechanism of phosphorus-based flame retardants, the phosphorus species decomposed from phytic acid may also yield phosphorus-containing radicals (e.g., •PO and •PO_2_) during thermal decomposition [39,40]. These species may work synergistically with the fluorine-containing radicals to jointly scavenge the active free radicals in flame, further strengthening the radical quenching effect to achieve chemical inhibition. It should be noted that the proposed chemical inhibition mechanism is inferred from the present results and relevant literature, and the direct experimental evidence for the radical-scavenging species is beyond the scope of the present study.

In addition, PA-MEL@PU@CFO microcapsules in this study exhibit superior fire suppression performance compared with the single-shell PU@CFO microcapsules and the previously reported MF@PU@CFO microcapsules. The PA-MEL shell is suggested to contribute to flame retardancy through phosphorus–nitrogen synergistic charring, which is mainly responsible for the superior fire suppression performance of PA-MEL@PU@CFO microcapsules. In contrast, the single-shell PU@CFO microcapsules may achieve fire suppression mainly by releasing the encapsulated CFO, which functions through physical cooling and gas-phase radical quenching [41]. MF resin merely provides single gas-phase flame retardant mechanism [42]. As a high-efficiency charring agent, PA groups in the shell structure decomposes at high temperatures to yield phosphoric acid (H_3_PO_4_), metaphosphoric acid (HPO_3_), and polyphosphoric acid (H_n+2_P_n_O_3n+1_). The acidic substances act as strong dehydration catalysts to accelerate the dehydration and carbonization of polymer shell matrix, rapidly constructing a dense and continuous phosphoric acid carbon layer on the surface of microcapsules [43]. Meanwhile, MEL and PU groups in the structure decompose upon heating and produce NH_3_, N_2_, CO_2_ and other inert non-combustible gases. The released inert gases penetrate into the molten phosphoric acid carbon layer, generating a porous yet compact intumescent carbon layer. The intumescent carbon layer can effectively isolate external heat, oxygen and combustible volatiles, thus restraining the continuous thermal degradation of the polymer shell matrix. Simultaneously, the intumescent carbon layer delays premature leakage of the encapsulated CFO, enabling the targeted and concentrated release of fire extinguishing agent at the critical fire stage. Consequently, PA-MEL@PU@CFO microcapsules exhibit superior fire suppression performance than the conventional resin-coated microcapsules.

## 4. Conclusions

In this study, novel double-shell PA-MEL@PU@CFO microcapsules were successfully prepared and their fire suppression performance in confined spaces was investigated in a simulated confined space experimental device. The main conclusions are as follows:

(i) Facile preparation of novel double-shell PA-MEL@PU@CFO microcapsules was developed. The single-shell PU@CFO microcapsules were prepared through interfacial polymerization method using CFO as the core material, PU generated from the polymerization of IPDI and DETA as the inner shell. The double-shell PA-MEL@PU@CFO microcapsules were then fabricated by further coating the as-prepared PU@CFO microcapsules with the outer shell of PA-MEL, which was synthesized by crosslinking reaction of PA and MEL.
(ii) Effects of the emulsification rotation on the morphology of microcapsules were investigated in detail. The optimal emulsification conditions for preparing single-shell PU@CFO microcapsules were determined as emulsification rotation speed of 3000 rpm, emulsification time of 10 min, IPDI/DETA monomer ratio of 1:2.5, and CFO loading amount of 4 mL. PU@CFO microcapsules fabricated under the optimal emulsification conditions exhibited regular spherical morphology with smooth surface and uniform particle size.
(iii) The combined characterization results demonstrated that the double-shell structure was successfully constructed for PA-MEL@PU@CFO microcapsules. SEM images revealed that PA-MEL@PU@CFO exhibited a regular spherical morphology with rough flocculent surface and uniform particle size of approximately 2.0 μm. TG/DTG analysis results demonstrated that the introduction of the outer PA-MEL shell raised the temperature for core leakage by 30 °C, decreased the decomposition rate of the inner PU shell and increased the final char yield of microcapsules. FT-IR spectra exhibited characteristic peaks of the inner shell of PU and the outer shell of PA-MEL, further confirming the successful coating. PA-MEL@PU@CFO microcapsules in this work exhibited overall favorable storage stability with a superior core content of 53.18%.
(iv) PA-MEL@PU@CFO microcapsules exhibited excellent flame retardant and fire-extinguishing performance for the fires in confined spaces at the incipient fire stage, reducing the maximum flame temperature by 238.6 °C, shortening the extinguishing time by 53 s, and effectively inhibiting flame re-ignition. PA-MEL@PU@CFO microcapsules achieved efficient fire suppression by a synergistic effect of physical cooling, chemical suppression and phosphorus–nitrogen synergistic flame retardancy.

This study proposes a facile preparation strategy for the fabrication of a novel double-shell PA-MEL@PU@CFO microcapsules for fire suppression in confined spaces. The combined results indicate that PA-MEL@PU@CFO microcapsules exhibit excellent fire suppression performance and possess great potential for industrial application. Follow-up studies will concentrate on further optimization of the fabrication parameters for higher core content and controllable core release, systematically investigating the long-term storage stability and fire suppression performance in practical application such as anti-flame coatings and fire safety protection of lithium-ion batteries and electrical equipment.

## Figures and Tables

**Figure 1:**
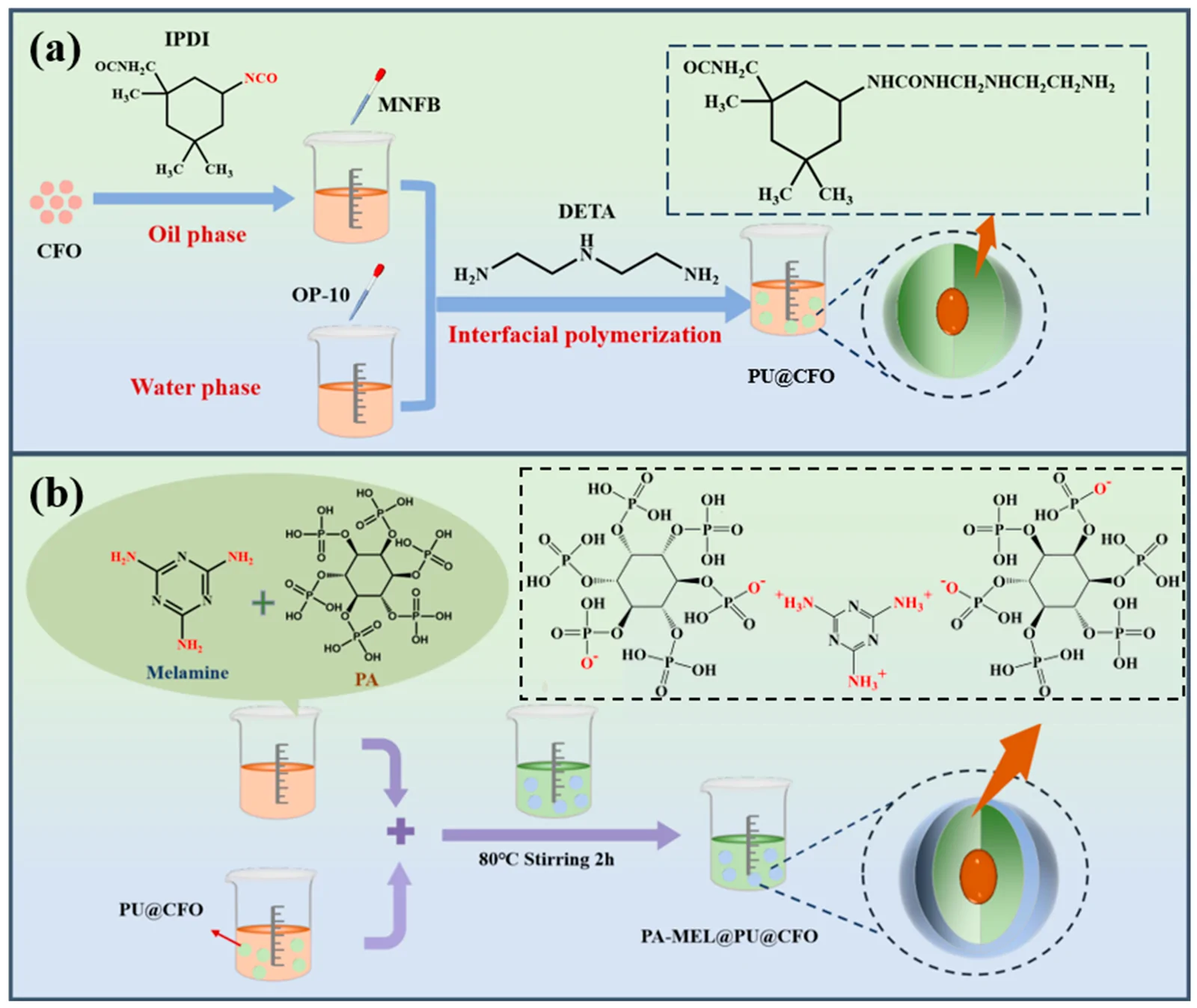
Preparation process of (**a**) PU@CFO and (**b**) PA-MEL@PU@CFO.

**Figure 2:**
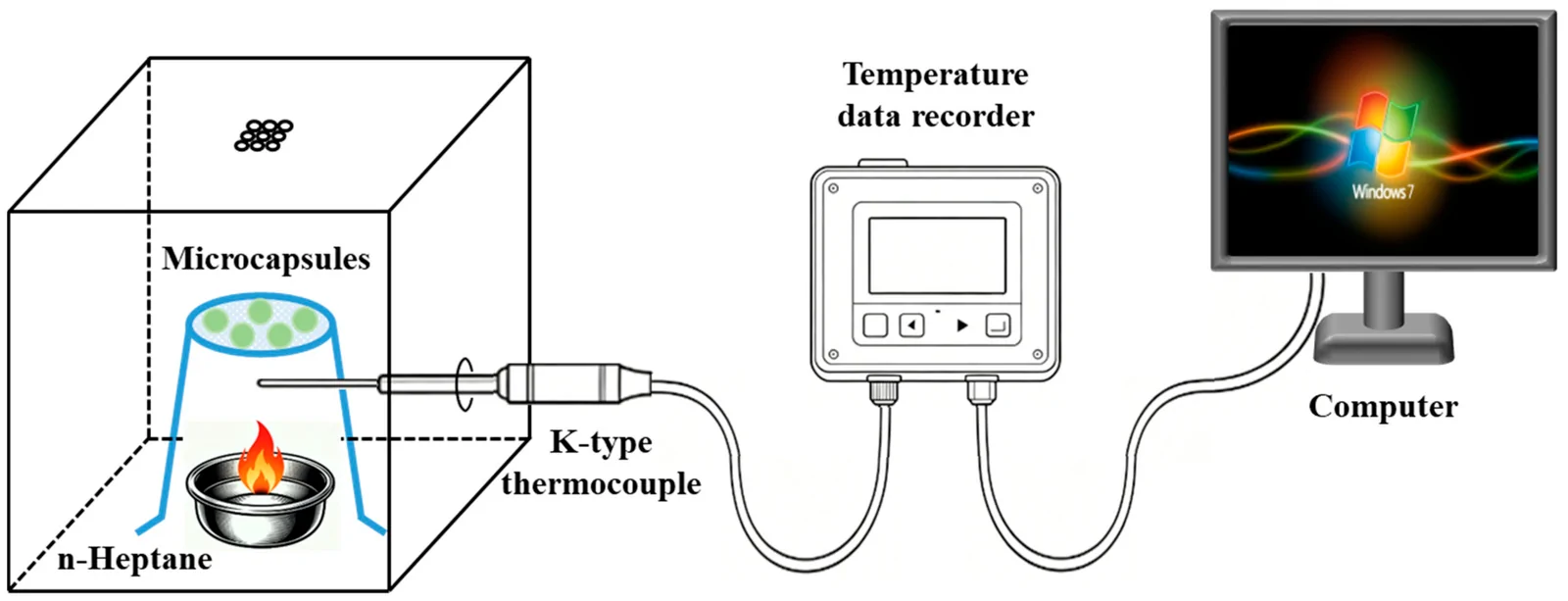
Apparatus schematic diagram fire suppression performance test.

**Figure 3:**
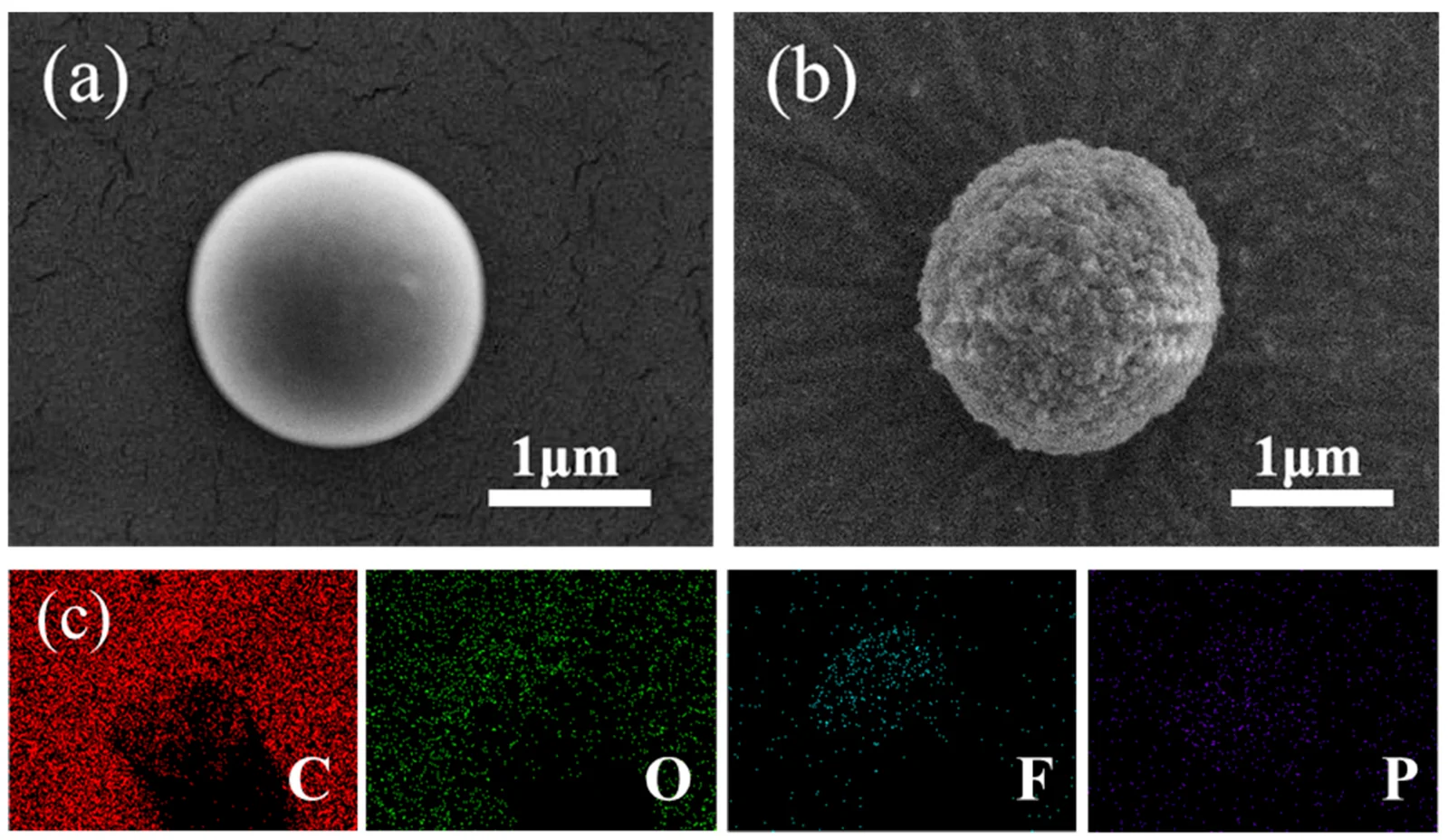
SEM images and EDS elemental mappings of microcapsules. (**a**) SEM image of PU@CFO, (**b**) SEM image and (**c**) EDS elemental mappings of PA-MEL@PU@CFO.

**Figure 4:**
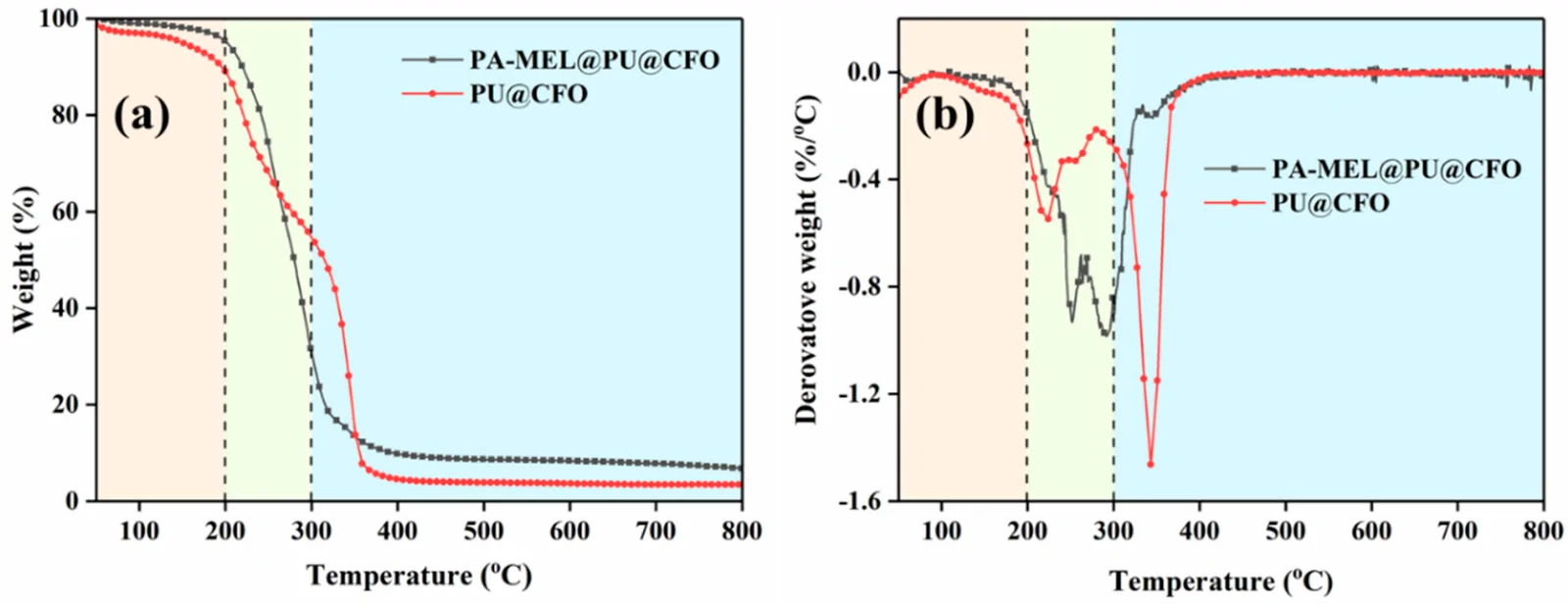
(**a**) TG and (**b**) DTG curves of PU@CFO and PA-MEL@PU@CFO.

**Figure 5:**
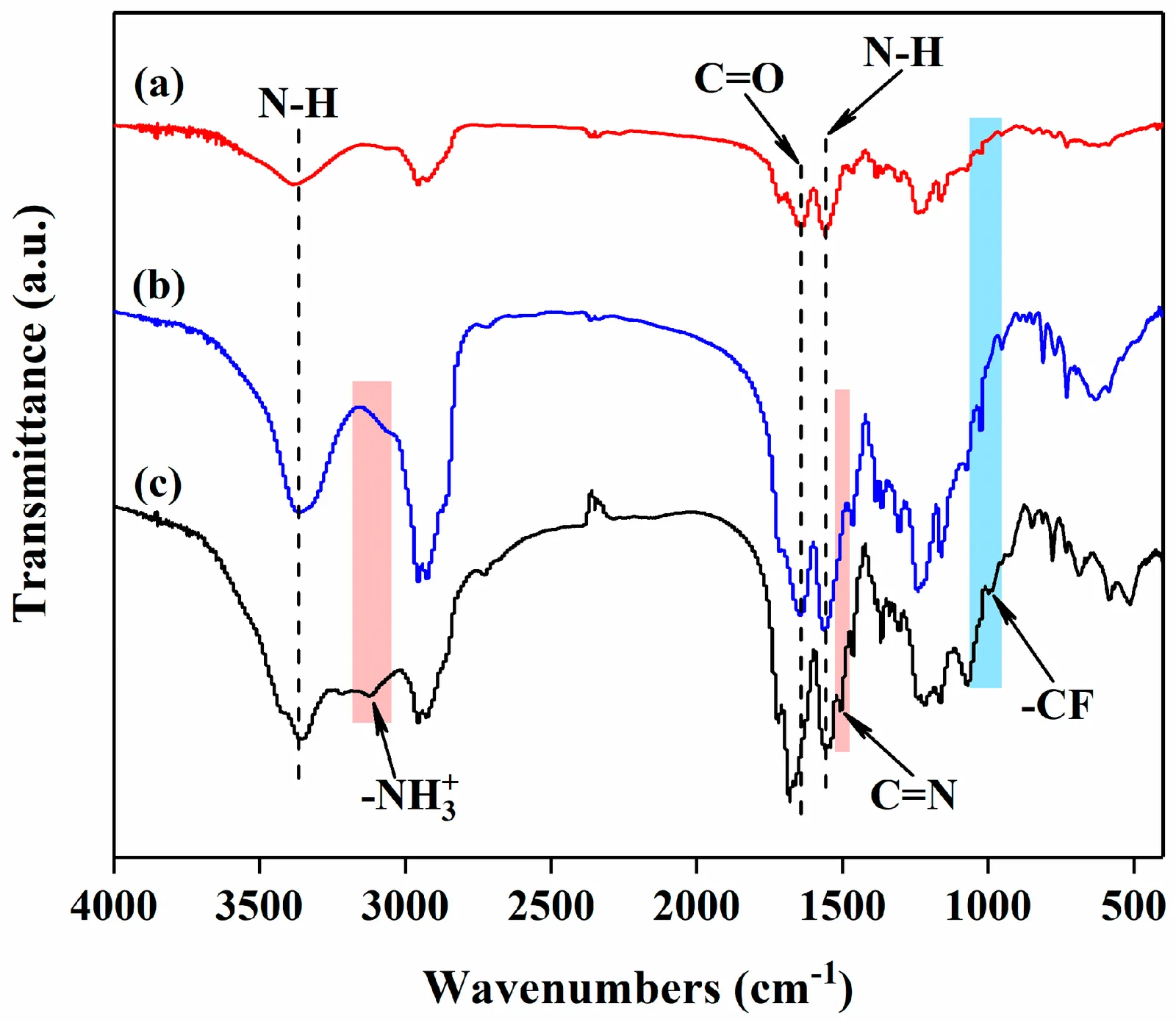
FT-IR spectra of (**a**) PU shell, (**b**) PU@CFO and (**c**) PA-MEL@PU@CFO.

**Figure 6:**
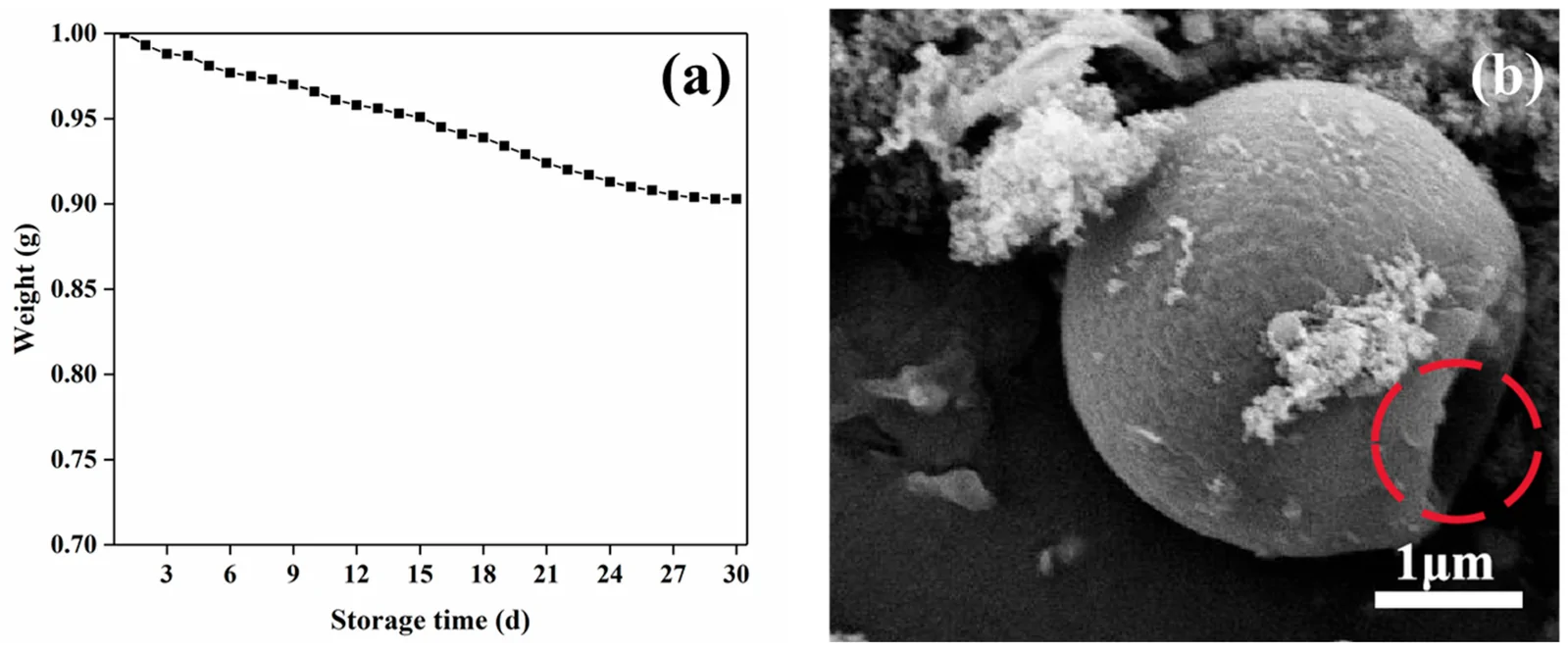
Storage stability of PA-MEL@PU@CFO. (**a**) Weight change during 30-day storage, (**b**) SEM image of aged PA-MEL@PU@CFO after 30-day storage test.

**Figure 7:**
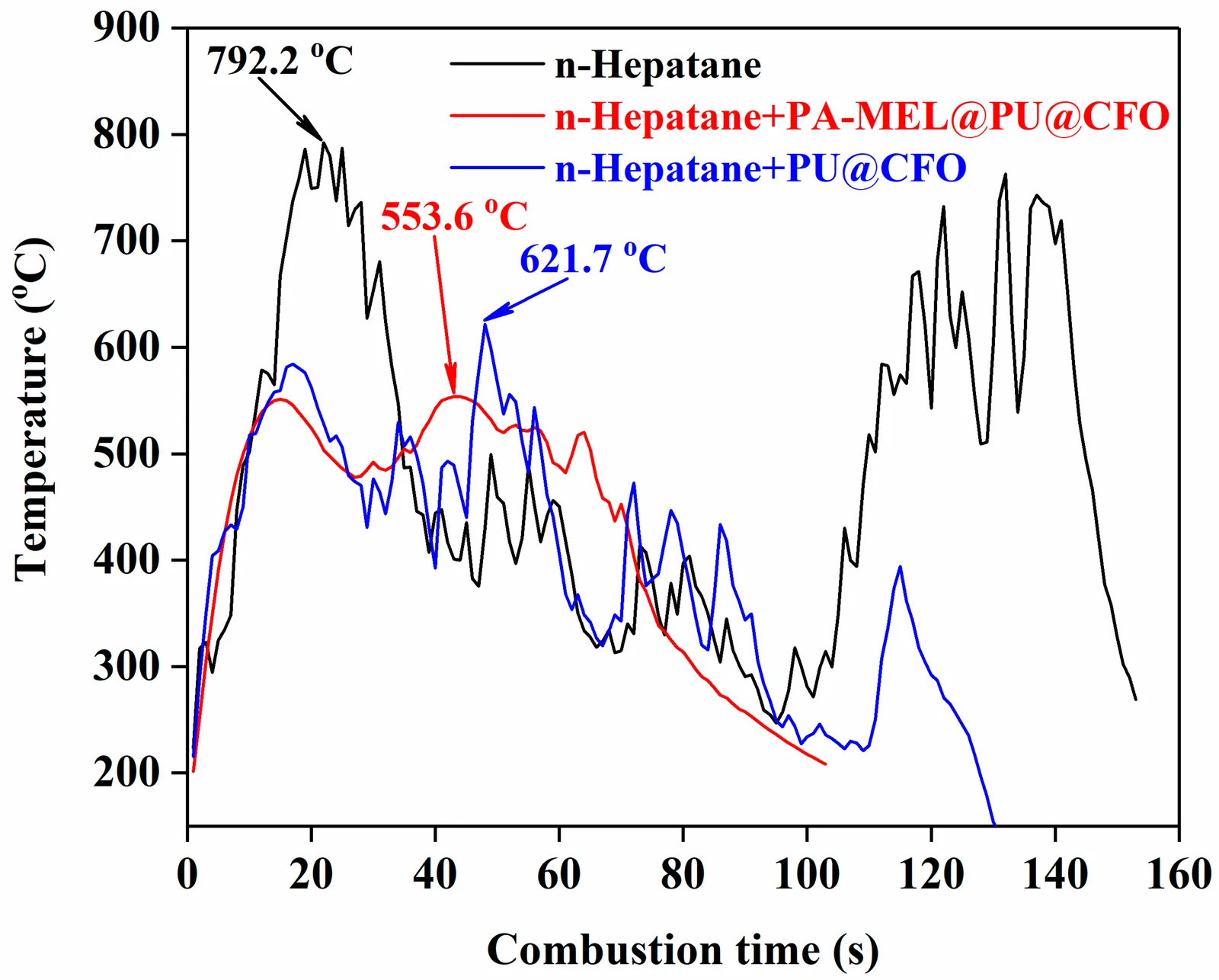
Temperature curves in fire suppression performance test of PA-MEL@PU@CFO.

**Figure 8:**
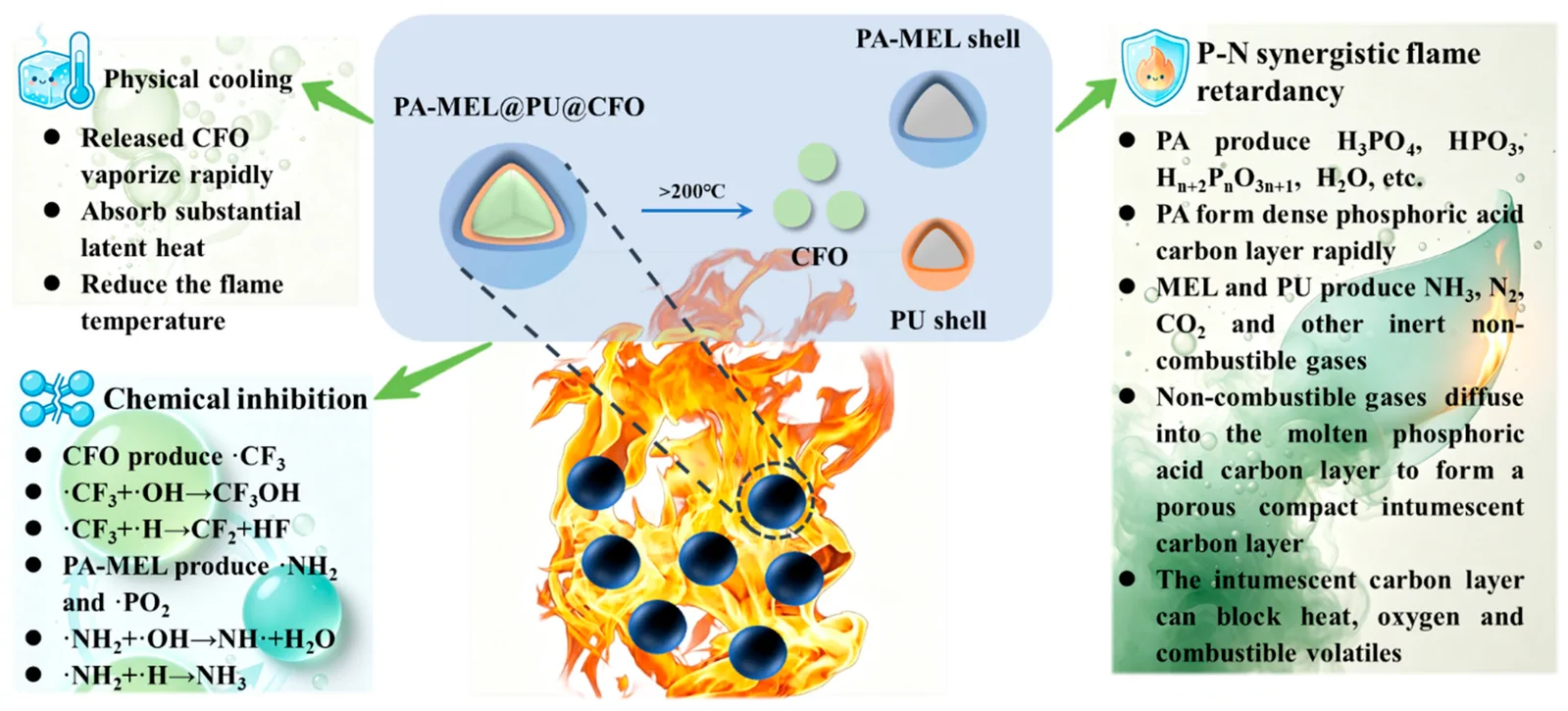
Schematic diagram of the proposed fire suppression mechanism of PA-MEL@PU@CFO.

**Table 1:** Fire suppression performance test results.

Material	Maximum Flame Temperature/°C	Time to Achieve Maximum Temperature/s	Extinguishing Time/s
n-Heptane	792.2	22	159
n-Heptane + PU@CFO	621.7	48	134
n-Heptane + PA-MEL@PU@CFO	553.6	43	106
n-Heptane + MF@PU@CFO [24]	520.2	40	123

## Data Availability

The original contributions presented in this study are included in the article/Supplementary Materials. Further inquiries can be directed to the corresponding authors.

## Supplementary Materials

The following supporting information can be downloaded at: https://www.mdpi.com/article/10.3390/ma19194135/s1, Figure S1: SEM images of PU@CFO microcapsules prepared at different emulsification rotation speeds of (**a**) 2000 rpm, (**b**) 3000 rpm, (**c**) 4000 rpm, and (**d**) 5000 rpm; Figure S2: TG and DTG curves of PU@CFO microcapsules prepared at different emulsification rotation speeds of (**a**) 2000 rpm, (**b**) 3000 rpm, (**c**) 4000 rpm, and (**d**) 5000 rpm; Figure S3: SEM images of PU@CFO microcapsules prepared at different emulsification times of (**a**) 3 min, (**b**) 5 min, (**c**) 10 min, and (**d**) 15 min; Figure S4: TG and DTG curves of PU@CFO microcapsules prepared at different emulsification times of (**a**) 3 min, (**b**) 5 min, (**c**) 10 min, and (**d**) 15 min; Figure S5: SEM images of PU@CFO microcapsules prepared at different IPDI/DETA monomer ratios of (**a**) 1:2, (**b**) 1:2.5, (**c**) 1:3, and (**d**) 1:3.5; Figure S6: TG and DTG curves of PU@CFO microcapsules prepared at different IPDI/DETA monomer ratios of (**a**) 1:2, (**b**) 1:2.5, (**c**) 1:3, and (**d**) 1:3.5; Figure S7: SEM images of PU@CFO microcapsules prepared at different CFO loading amounts of (**a**) 2 mL, (**b**) 4 mL, (**c**) 6 mL, and (**d**) 8 mL; Figure S8: TG and DTG curves of PU@CFO microcapsules prepared at different CFO loading amounts of (**a**) 2 mL, (**b**) 4 mL, (**c**) 6 mL, and (**d**) 8 mL; Figure S9: FT-IR spectra of PA-MEL@PU@CFO and the residual sample after grinding and drying at 120 °C for 12 h.

## References

[1] Li Y., Wan X., Jiang N. (2026). A Review on Recent Advances, Progressive Design, and Future Challenges of Separators in Lithium-Ion Batteries. Energy Technol..

[2] Chen W., Yang H., Yue N., Yan X., Wu T., Peng C. (2026). On the Sustainability of Lithium-Ion Battery-Powered Electric Vehicles: Critical Review, Analysis, and Perspectives. Energy Environ. Mater..

[3] Mohyeldien O.M., El-Amary N.H., Al Bardawil A. (2026). Next-generation lithium-ion batteries: A comparative review based on advanced materials, electrolyte innovations, and future perspectives. J. Power Sources.

[4] Zhang H., Zhao C., Zhang Z., Liu J., Panchal S., Xu X., Zhou F. (2026). Review on the thermal management and thermal suppression of high-energy-density lithium-ion batteries based on energy-efficient liquid cooling techniques. Appl. Therm. Eng..

[5] Chen Z., Zhang J., Liu C., Yang C., Chen S. (2026). Thermal Runaway in Lithium-Ion Batteries: A Review of Mechanisms, Prediction Approaches, and Mitigation Strategies. Batteries.

[6] Wang Y., Xiong G., Zeng D., Mohaddes D. (2026). Towards fire hazard evaluation of stationary lithium-ion battery systems: A review. Fire Saf. J..

[7] Yao L., Yu C., Xiao Y., Cui G., Fei Z., Qu C. (2025). A comprehensive review of lithium-ion battery safety issues and fault diagnosis strategies throughout the entire lifecycle. J. Energy Storage.

[8] Liu H., Zhang T., Zhang M., Zhang C., Guo Z., Zhang Y., Chen H., Wu Y., Zhang G. (2024). Preparation and thermal responsiveness of microencapsulated fluorinated liquids for automatic fire extinguishing. Heliyon.

[9] Li C., Bian H., Ding D., Huang F., Zhu Z. (2024). Enhancing safety in small confined spaces with thermally triggered fire-extinguishing microcapsules from microfluidics. Lab Chip.

[10] Meng X., Wang Z., Liu B., Gao Y., Zhang J., Sun J., Wang Q. (2024). Enhancing extinguishing efficiency for lithium-ion battery fire: Investigating the extinguishing mechanism and surface/interfacial activity of F-500 microcapsule extinguishing agent. eTransportation.

[11] Fadini A.L., Alvim I.D., Ribeiro I.P., Ruzene L.G., da Silva L.B., Queiroz M.B., Miguel A.M.R.D., Chaves F.C.M., Rodrigues R.A.F. (2018). Innovative strategy based on combined microencapsulation technologies for food application and the influence of wall material composition. LWT-Food Sci. Technol..

[12] Wang X., He Z., Gao J., Guo Y., Zhang H., Wang M. (2025). External Barrier and Internal Attack: Synergistic Effect of Microcapsule Fire Extinguishing Agent and Fine Water Mist on Suppressing Lithium-Ion Battery Fire. Materials.

[13] Yim T., Park M.S., Woo S.G., Kwon H.K., Yoo J.K., Jung Y.S., Kim K.J., Yu J.S., Kim Y.J. (2015). Self-Extinguishing Lithium Ion Batteries Based on Internally Embedded Fire-Extinguishing Microcapsules with Temperature-Responsiveness. Nano Lett..

[14] Huang J., Jin J., Liang J., He Y., Chen Y. (2025). Experimental study on fire suppression of NCM lithium-ion battery by C_6_F_12_O in a confined space. Appl. Therm. Eng..

[15] Yang Q., Gao Y., Yang Y., Zhou X., Zhang H. (2024). Toward better halon substitutes: Effects of carbon chain length on pyrolytic and fire-suppressing mechanisms of perfluoroalkanes. J. Mol. Struct..

[16] Liu Q., Yang R., Zhu P. (2025). Study on the Effectiveness of Perfluorohexanone in Extinguishing Small-Scale Pool Fires in Enclosed Compartments Under Low-Pressure Conditions. Fire.

[17] Yu W., Zhang T., Guo Z., Liu H., Yuan J. (2025). Simulation study on the thermal response characteristics of the encapsulated perfluorohexanone extinguishing agent. Case Stud. Therm. Eng..

[18] Pan Q., Sang N., Zhou T., Wu C., Si T., Huang F., Zhu Z. (2025). Array-structured microcapsule fibers for efficient fire extinguishing in confined spaces. Lab Chip.

[19] Tian S., Yao C., Huang Z., Maluta N., Yang M., Zhang X. (2026). In-situ polymerized B_4_C-MUF@perfluoro(2-methyl-3-pentanone) microcapsules for enhanced thermal stability and synergistic flame retardancy. Polym. Degrad. Stab..

[20] Han Z., Li Z., Yang J. (2025). Highly effective suppression of accidental combustion and explosion on B/KNO_3_ by perfluorohexanone microcapsule. Fuel.

[21] Xiao Y., Pan Y., Yang J., Li L., Zhao Z., Cheng X., Zhang H. (2025). Enhanced safety of lithium-ion batteries by a novel bifunctional aerogel sheet coated with fire extinguishing microcapsules. Chem. Eng. J..

[22] Meng H., Zhang T., Wang Z., Kong L., Wei Z., Dong W., Li D., Liang H., Zhang C. (2025). Experimental study on a novel safety strategy for lithium-ion batteries: Fire suppression and thermal runaway mitigation in confined spaces using heat-triggered Fire-extinguishing Polymer Composite (FEPC). Chem. Eng. J..

[23] Guo Y., Wang X., Gao J., He Z., Yao S., Zhou X., Zhang H. (2024). In situ extinguishing mechanism and performance of self-portable microcapsule fire extinguishing agent for lithium-ion batteries. J. Energy Storage.

[24] Xue H., Wang Y., Ou H., Zhou W., Su J., Wen Z. (2026). Preparation of MF@PU@CFO microcapsules and fire extinguishing performance tests. Int. J. Polym. Anal. Charact..

[25] Song W., Zhang L., Wang B., Liu Y. (2026). Integrating reusability with multifunctionality: An eco-friendly phytic acid-tannic acid finishing agent for flame retardant, antibacterial, and UV protective Lyocell fabric. Bioresour. Technol..

[26] Wang S., Yang Y., Zhang L., Liu H., Ma H. (2026). Supramolecular Phytic Acid/Melamine Self-Assembly Coatings for Flexible Polyurethane Foam with Superior Flame Retardancy and Fire-Warning. J. Appl. Polym. Sci..

[27] Huang H., Yan W., Tan S., Zhao Y., Dong H., Liao W., Shi P., Yang X., He Q. (2024). Frontier in gellan gum-based microcapsules obtained by emulsification: Core-shell structure, interaction mechanism, intervention strategies. Int. J. Biol. Macromol..

[28] Song X., Yu Y., Yang G., Jiang A., Ruan Y., Fan S. (2022). One-step emulsification for controllable preparation of ethyl cellulose microcapsules and their sustained release performance. Colloid Surf. B.

[29] Muñoz-Adasme A., Navarrete A., Hermosilla-Dote E., Garcia-Herrera C., Aranda P., Zambra C., Vasco D.A. (2026). Effect of ultrasonication on the thermal and mechanical properties of plasterboard modified with a microencapsulated phase change material. J. Build. Eng..

[30] Song Y., Li J., Wang X., Song G., Li X., Cui Y., Feng Z., Ning Y., Wu Y. (2024). Grafting isocyanate onto graphene oxide for polyurethane composites to improve their thermal stability and mechanical properties. Iran. Polym. J..

[31] Ni X., Chen Y., Huang Q., Zhao C., Li S., Huang J., Wang J. (2023). An experimental study on the transportation characteristics of perfluoro(2-methyl-3-pentanone) in a straight pipe. Fire.

[32] Wei A., Wang S., Zou Y., Xiang C., Xu F., Sun L. (2024). Preparation of a flame-retardant curing agent based on phytic acid-melamine ion crosslinking and its application in wood coatings. Polymers.

[33] Zheng X., Zou Y., Xiang C., Wei A., Wei Y., Sun L. (2025). Dual flame-retardant and curing-agent effects of phytic acid-guanazole as an additive in fire-protective coatings for wood. Polymers.

[34] Jin X., Tian S., Liu B., Yuan Z., Li X., Wang K., Yang M., Yang R. (2026). Fire Inhibition Mechanism of C_6_F_12_O on Li-Ion Battery Thermal Runaway Vent Gas: A Reactive Force-Field Molecular Dynamics Study. Energy Technol..

[35] Liu F., Bai Z., Zhang P., Liu Y., Chen B., Gao J. (2026). Preparation and release dynamics of perfluorohexanone thermosensitive microcapsules. J. Therm. Anal. Calorim..

[36] Jin Z., Zhang X. (2023). The fire-extinguishing performance and mechanism of fluorinated cyclobutane through experimental measurement and numerical calculation. New J. Chem..

[37] Wang L., Zhao H., Meng L., Chen H., Jia L. (2024). Preparation of phosphorus-nitrogen synergistic flame retardant cellulose composite aerogel from waste cotton/phytic acid/acrylamide. Int. J. Biol. Macromol..

[38] Mishra B.K., Lily M., Deka R.C., Chandra A.K. (2016). A theoretical insight into atmospheric chemistry of HFE-7100 and perfluoro-butyl formate: Reactions with OH radicals and Cl atoms and the fate of alkoxy radicals. New J. Chem..

[39] Zheng Y., Jiang Z., Wu T. (2025). The development and application of contemporary phosphorus flame retardants: A review. Front. Mater..

[40] Howell B. (2022). Thermal Degradation of Organophosphorus Flame Retardants. Polymers.

[41] Wen Z., Xiong L., Wang Y., Wang Z., Xue H., Tu Z. (2026). Preparation and Performance Investigation of a Novel Microcapsule Fire Extinguishing Agent for Confined Space Fire Suppression. J. Appl. Polym. Sci..

[42] Pan R., Yu E., Wang Y., Liu G., Wei Z. (2025). Phytic acid and melamine-modified microcrystalline cellulose as effective flame retardants in polylactic acid composites. Carbohyd. Polym..

[43] Dong S., Haung Y., Zhang X., Cheng S., Cheng X., Guan J. (2024). Enhancing the Flame Retardancy of Polyester/Cotton Blend Fabrics Using Biobased Urea-Phytate Salt. Materials.

