# Supplementary material for: Facile Fabrication of Double-Shell PA-MEL@PU@CFO Microcapsules and Their Fire Suppression Performance in Confined Spaces
Source: Materials (Basel). 2026 Sep 28;19(19):4135. doi: 10.3390/ma19194135 (PMC13666400; doi:10.3390/ma19194135)
Supplement: Supplementary file 1 [file materials-19-04135-s001.zip › materials-4566275-supplementary.pdf]

Supporting Information

Facile Fabrication of Double-Shell PA-MEL@PU@CFO Microcapsules and Their Fire Suppression Performance in Confined Spaces

Zhe Wen [1,2], Zihan Wang [3], Lingfeng Xiong [3], Hongxiang Ou [3], Fang Zhu [3], Honglai Xue [3,4,*] and Yingchu Wang [3,*]

[1] School of Petrochemical Engineering, Changzhou University, Changzhou 213164, China
[2] Jiangsu Province Key Laboratory of Fine Petrochemical Engineering, Changzhou University, Changzhou 213164, China
[3] School of Safety Science and Engineering, Changzhou University, Changzhou 213164, China
[4] State Key Laboratory of Fire Science, University of Science and Technology of China, Hefei 230026, China
* Correspondence: xuehonglai@cczu.edu.cn (H.X.); s23200857034@smail.cczu.edu.cn (Y.W.)

Section S1. Experimental

S1.1 Preparation of single-shell PU@CFO microcapsules

PU@CFO microcapsules were prepared through an interfacial polymerization method, in which CFO was used as the core material, polyurea (PU) derived from the in situ polymerization of IPDI and DETA was used as the wall material. The preparation process is schematically illustrated in Figure 1(a). Briefly, 4 mL of CFO, 0.64 g of IPDI and 0.64 g of MNFB were mixed homogeneously at room temperature to obtain the oil-phase mixture. 0.3 g of OP-10 was then dissolved in 30 mL of deionized water to obtain the water-phase solution. The oil-phase mixture was added dropwise into the water-phase solution under high-speed stirring at room temperature. The resulted oil-water mixture was continuously emulsified at 3000 rpm for 10 min. A homogeneous oil-in-water (O/W) emulsion was obtained and then transferred into a sealed tube and heated in a water bath. The polymerization reaction was initiated by adding a certain amount of DETA into the resulted O/W emulsion. The polymerization reaction was firstly carried out at room temperature for 1 h, then heated to 45 °C and held for another 2 h, at a stirring speed of 200-400 rpm. After the reaction, PU@CFO microcapsules were obtained after filtration, washing and freeze-drying.

S1.2 Determination of core content

The core content of microcapsules was determined according to the following procedures. An oven-dried microcapsule sample with a mass of M_1 was firstly ground into powder to rupture the shell structure and then transferred into a vacuum drying oven. The powder was heated at 120 °C for 12 h to achieve the complete release of the core materials. A pure shell

material was finally obtained and weighted with a mass denoted as M_2. The core content of microcapsules was calculated according to the following formula:

$$\text{Core content (\%)} = \frac{M_1 - M_2}{M_1} \times 100\%$$

S1.3 Evaluation of storage stability

The storage stability of microcapsules was evaluated via a 30-day storage test under ambient storage conditions. Briefly, freshly prepared microcapsules were stored under ambient conditions for 30 days. The weight of microcapsules was measured every day to evaluate the weight change, using freshly prepared microcapsules as the control. Meanwhile, the surface morphologies of the microcapsules after 30-day storage was observed by SEM.

Section S2. Effects of emulsification conditions on the morphology of PU@CFO

S2.1 Effect of emulsification rotation speed

Emulsification rotation speed is one of the core process parameters for the preparation of microcapsules, which directly affecting the morphology of microcapsules including size distribution, spherical integrity, and agglomeration degree. The effect of rotation speed on the morphology of microcapsules is mainly realized by changing the shear force, energy input and flow field state of the system. The effect of emulsification rotation speed on the morphology of PU@CFO was investigated at a fixed emulsification time of 10 min. The SEM images and TG curves of the final PU@CFO microcapsules obtained under different emulsification rotation speeds were presented in Figure S1 and Figure S2, respectively.

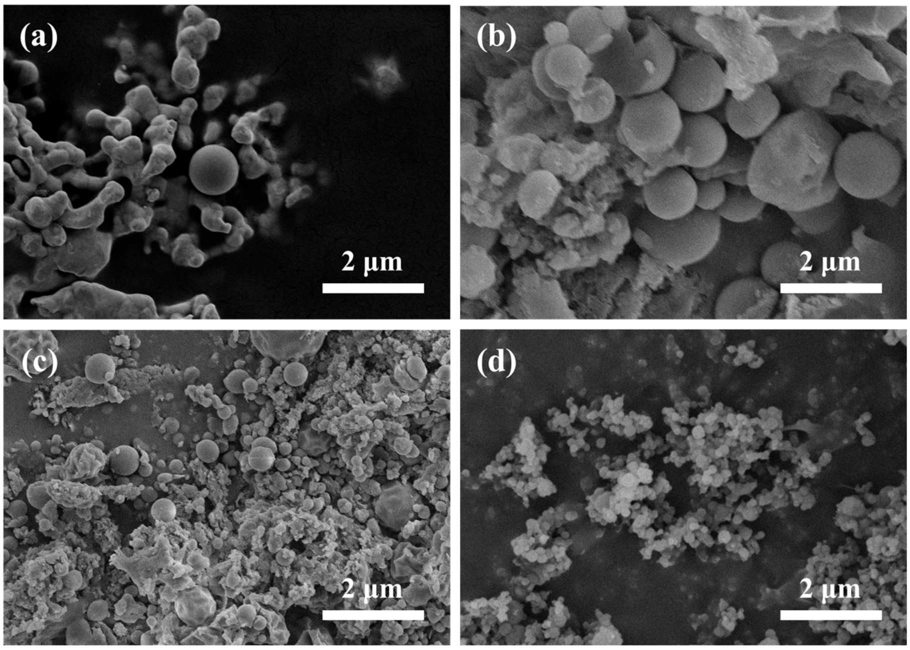

Figure S1. SEM images of PU@CFO microcapsules prepared at different emulsification rotation speeds of (a) 2000 rpm, (b) 3000 rpm, (c) 4000 rpm, and (d) 5000 rpm

At an emulsification rotation speed of 2000 rpm, almost no intact spherical microcapsules but a small number of irregular rod-like and dumbbell-like structures could be observed on the SEM micrograph shown in Figure S1(a). And severe agglomeration of wall materials was observed, along with heterogeneous particle size and poor dispersity. It was attributed to that the insufficient shear force at low rotation speeds was unable to fully stabilize the dispersion of the large oil phase droplets in the aqueous phase, resulting in the unstable nucleation and encapsulation processes of microcapsules. Generally, single-shell microcapsules exhibit two primary weight-loss stages during thermal decomposition, attributed to the leakage of core materials and the decomposition of wall materials, respectively. However, three distinct peaks were observed in the DTG curve of PU@CFO microcapsules obtained at emulsification rotation speed of 2000 rpm, as shown in Figure S2(a). This phenomenon was ascribed in the insufficient emulsification at low rotation speeds. In addition to the as-prepared microcapsules

characterized with non-uniform particle sizes and irregular morphology, residual pure wall materials were also present in the sample. Multiple weight-loss peaks were consequently obtained due to the significant differences in the thermal decomposition temperatures of various components. The three distinct weight-loss peaks in the DTG curve of PU@CFO were assigned to the leakage of core materials, the decomposition of wall materials, and the thermal degradation of agglomerated pure shell particles, respectively.

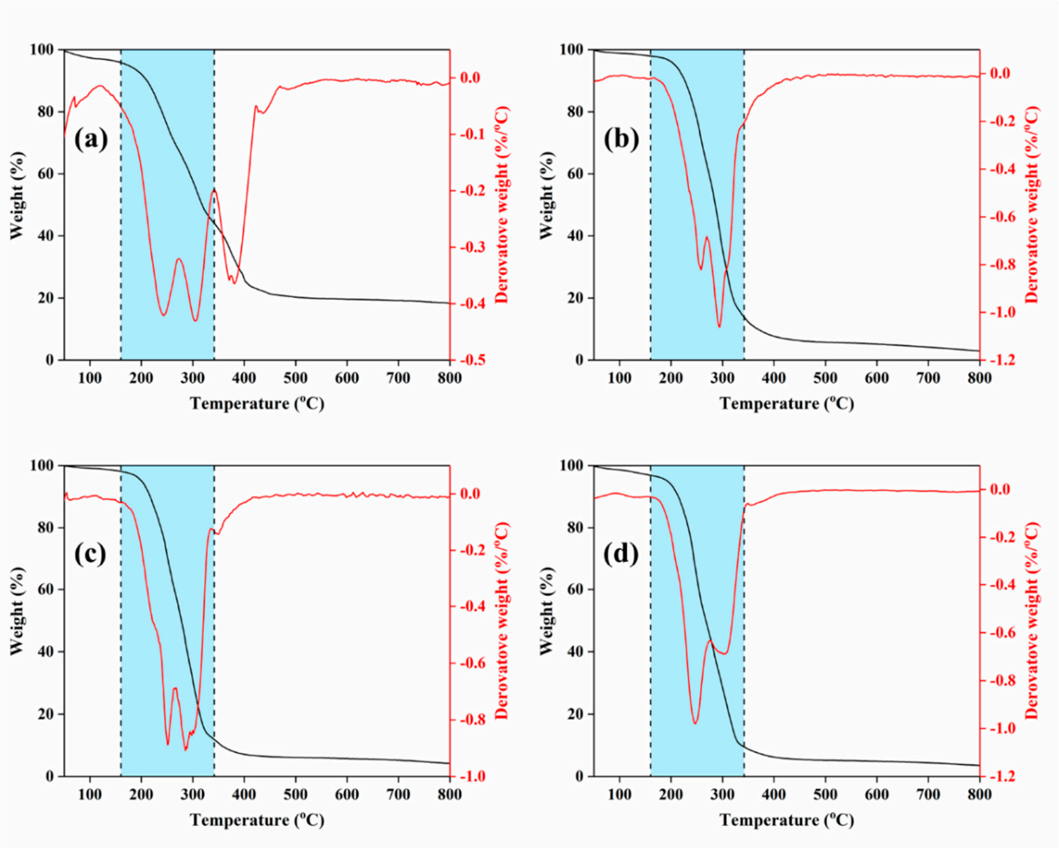

Figure S2. TG and DTG curves of PU@CFO microcapsules prepared at different emulsification rotation speeds of (a) 2000 rpm, (b) 3000 rpm, (c) 4000 rpm, and (d) 5000 rpm

As shown in Figure S1(b), when the emulsification rotation speed was increased to 3000 rpm, most PU@CFO microcapsules exhibited regular spherical morphology with smooth surface and uniform particle size. No obvious adhesion or agglomeration occurred in the

system and the dispersity of microcapsules was significantly improved. As the rotation speed further increased to 4000 rpm and 5000 rpm, the dispersity of microcapsules instead became worse. This phenomenon was attributed to the excessive heat accumulation inside the system at ultra-high rotation speeds, which accelerated the rapid hydrolysis of IDPI and the intense volatilization of CFO. Meanwhile, excessive shear force would fracture the already formed core material droplets, resulting in poor core content and uneven particle size distribution of microcapsules. Self-agglomeration of wall material particles and severe adhesion between microcapsules could be observed, especially at ultra-high rotation speed of 5000 rpm. As shown in Figure S2(d), the mass loss rate of core materials was much higher than that of wall materials. The prominent low-temperature weight-loss peak further confirmed the poor core content of microcapsules at ultra-high rotation speeds, leading to massive and rapid volatilization of core materials. As concluded, an emulsification rotation speed of 3000 rpm was determined as the optimal emulsification rotation speed in our study.

S2.2 Effect of emulsification time

Emulsification time is also one of the core process parameters for microcapsules preparation, which will significantly affect the dispersion state of the emulsion by regulating the interfacial behavior and the energy state of the emulsion system. The effect of emulsification time on the morphology of PU@CFO was investigated at a fixed emulsification rotation speed of 3000 rpm. The SEM images and TG curves of the final PU@CFO microcapsules obtained under different emulsification times were exhibited in Figure S3 and Figure S4, respectively.

As shown in Figure S3(a), only a small number of imperfect spherical microcapsules with relatively large particle size could be observed in the SEM images of the final PU@CFO microcapsules obtained at emulsification time of 3 min. Some microcapsules possessed rough surfaces with cracks and irregular protrusions, indicating poor encapsulation integrity. Meanwhile, a large quantity of unreacted wall materials agglomerated together to form large irregular particles scattered around the microcapsules, resulting in disordered overall morphology. The DTG curve in Figure S4(a) showed that the characteristic peaks corresponding to core materials leakage were inconspicuous, indicating that microcapsules prepared at emulsification time of 3 min possessed poor core content. The mass loss of the samples was primarily contributed to the thermal decomposition of wall materials. As shown in Figure S3(b), a small number of individual microcapsules with intact spherical morphology could be observed as the emulsification time extended to 5 min, yet the overall number of microcapsules was still relatively low and the particle size was still relatively large. A large number of irregular agglomerated wall materials particles were still scattered around the microcapsules. However, microcapsules prepared at emulsification time of 5 min exhibited distinctly different thermal weight-loss characteristics from those prepared at emulsification time of 3 min. Multiple peaks could be observed on the DTG curve in Figure S4(b). This phenomenon was attributed to the insufficient emulsification at short emulsification time, resulting in microcapsules characterized with non-uniform particle sizes and uneven shell thickness. In addition, a large number of microcapsules with empty structure and microcapsule-in-microcapsule structure also appeared in the system. The difference in shell rupture

temperatures among various microcapsule structures resulted in the appearance of multiple weight-loss peaks. As concluded, insufficient emulsification time leads to emulsion droplets with non-uniform sizes due to the insufficient shear force. The emulsion droplets cannot be fully covered by emulsifiers and will easily collide and coalesce in the subsequent stirring process. As a result, the stability of emulsion is reduced. Microcapsules with drastically non-uniform particle sizes will be formed after the subsequent interfacial polymerization, accompanied by a large amount of free and agglomerated pure shell particles.

As shown in Figure S3(c), the quantity of microcapsules was significantly increased at emulsification time of 10 min. Most microcapsules displayed regular spherical morphology with smooth surface, uniform particle size, and negligible shell defects, achieving the best morphological integrity of microcapsules. The microcapsules were well dispersed without obvious adhesion or agglomeration, along with greatly decrease of irregular agglomerated wall materials particles. The DTG curve of microcapsules prepared at emulsification time of 10 min demonstrated in Figure S4(c) exhibited two distinct weight-loss peaks, corresponding to the leakage of core materials and the decomposition of wall materials, respectively. Appropriate emulsification time can provide sufficient shear force without excessive disturbance to the liquid-droplet interface, thereby generating fine emulsion droplets with uniform particle size. The emulsifier molecules orient at the oil-water interface to form a stable interfacial film outside the droplets, which can effectively prevent the adhesion or agglomeration of emulsion droplets. As a result, microcapsules with regular spherical morphology, uniform particle size and high core content can be obtained after subsequent polymerization reaction.

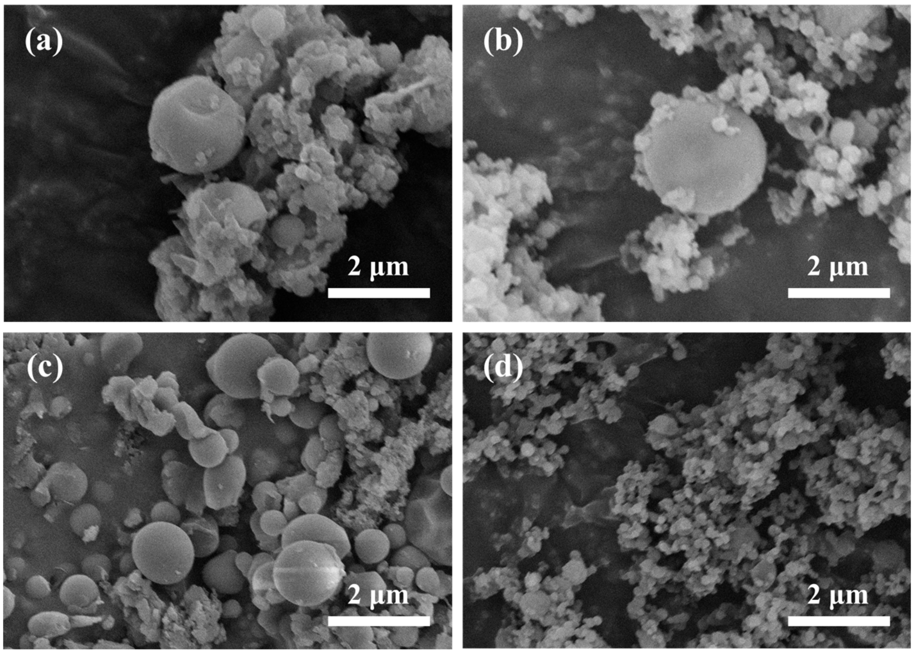

Figure S3. SEM images of PU@CFO microcapsules prepared at different emulsification times of (a) 3 min, (b) 5 min, (c) 10 min, and (d) 15 min

As the emulsification time further extended to 15 min, the dispersity of microcapsules instead became worse. A large amount of porous, flocculent, and irregular agglomerated wall materials particles but almost no well-formed microcapsules could be observed in the SEM images shown in Figure S3(d). When the emulsification time is excessively prolonged, the emulsification droplets tend to collide frequently and agglomerate with each other under continuous stirring, widening the particle size distribution of emulsification again. Meanwhile, the -NCO groups in IPDI can slowly undergo hydrolysis reaction in water during prolonged emulsification time. The already formed oil-water interfacial film will be disrupted for the weakened stabilizing emulsification effect of emulsifier, making the subsequent interfacial polymerization hard to proceed steadily. As a result, the encapsulation integrity of the as-prepared microcapsules decreases significantly, accompanied by deteriorated storage stability and sustained-release performance. As shown in Figure S4(d), the weight-loss peaks

corresponding to the leakage of core materials were relatively weak in the DTG curve of the microcapsules fabricated at 15 min, further indicating poor core content of microcapsules at excessively long emulsification time. As concluded, either excessively short or long emulsification time will deteriorate the formation performance and core content of microcapsules. The emulsification time of 15 min was determined as the optimal emulsification time in our study.

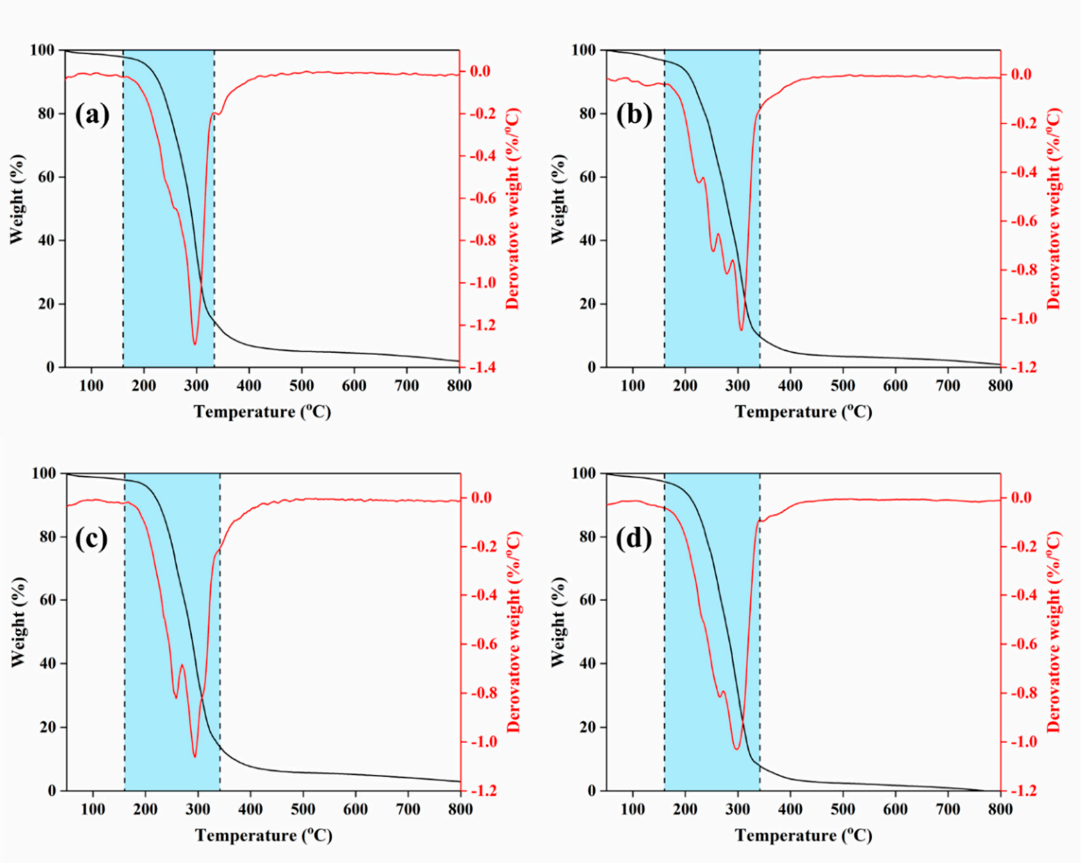

Figure S4. TG and DTG curves of PU@CFO microcapsules prepared at different emulsification times of (a) 3 min, (b) 5 min, (c) 10 min, and (d) 15 min

S2.3 Effect of IPDI/DETA monomer ratio

The ratio of IPDI to DETA, which are the fundamental monomers constituting the microcapsule wall, has significant influence on the core content, release performance and

storage stability of the as-prepared microcapsules. The effect of IPDI/DETA monomer ratio on the morphology of microcapsules was investigated at a fixed emulsification rotation speed of 3000 rpm and emulsification time of 10 min. The SEM images and TG curves of the final PU@CFO microcapsules obtained at different IPDI/DETA monomer ratios were presented in Figure S5 and Figure S6, respectively.

When IPDI/DETA monomer ratio was 1:2, almost no intact spherical microcapsules could be observed on the SEM micrograph shown in Figure S5(a). A large number of irregular dumbbell-like particles were instead generated in the sample system. The ratio of wall material monomers significantly affects the compactness and mechanical strength of the microcapsule shell by determining the degree of cross-linking reaction of monomers. Excessive -NCO groups from IPDI along with insufficient -NH_2 groups form DETA exist in the system at low IPDI/DETA monomer ratio. The unreacted -NCO groups can slowly undergo hydrolysis reaction and the generated new -NH_2 groups tend to further react with the residual -NCO groups to form low molecular weight by-products. Meanwhile, free -NCO groups can react with -NH groups from the already formed urea linkages to generate biuret. As a result, the initially formed shell exhibits poor compactness and low mechanical strength, which cannot provide effective protection for core droplets. The shells are prone to rupture under rapid stirring and frequent particle collision, further leading to the severe agglomeration of microcapsules. Multiple weight-loss peaks could be observed on the DTG curve in Figure S6(a), further indicating the poor core content at low IPDI/DETA monomer ratio.

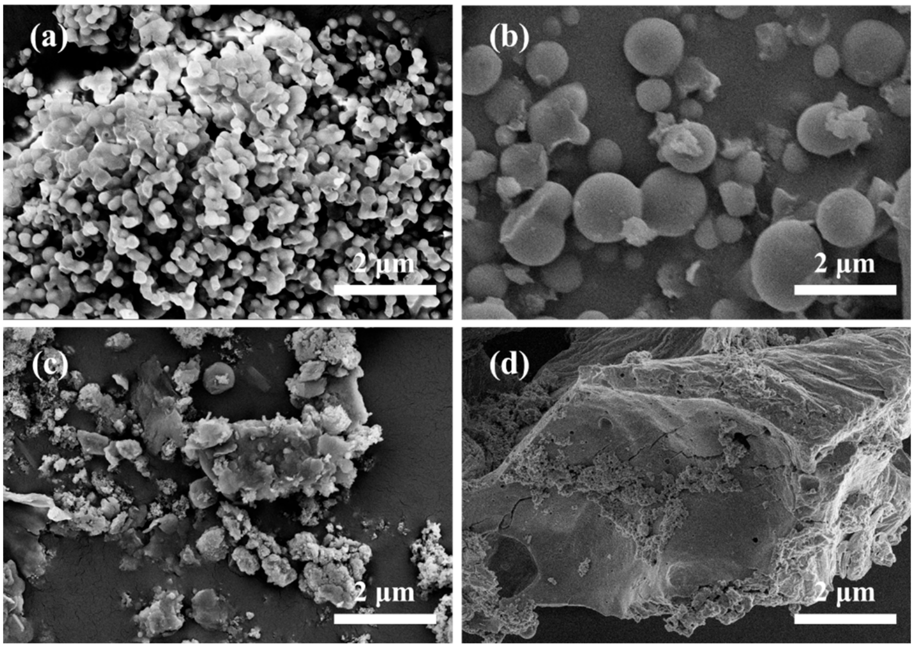

Figure S5. SEM images of PU@CFO microcapsules prepared at different IPDI/DETA monomer ratios of (a) 1:2, (b) 1:2.5, (c) 1:3, and (d) 1:3.5

As shown in Figure S5(b), appropriately increasing the dosage of DETA to IPDI/DETA monomer ratio of 1:2.5, most PU@CFO microcapsules exhibited a well-defined spherical morphology with smooth surface, uniform particle size, and compact shell. No obvious adhesion or agglomeration could be observed. The microcapsules were well dispersed with relatively uniform particle size of around 1-2 μm. Increased active amino groups in the system can facilitate sufficient crosslinking between -NCO and $-NH_2$ groups. Free isocyanate groups remained in the system can be maximally consumed, thus restraining the formation of biuret and some other low molecular weight by-products. The generated shell characterized by perfect compactness and high mechanical strength can stably encapsulate the core droplets, resisting shell rupture and particle agglomeration under stirring. The DTG curve of microcapsules prepared at IPDI/DETA monomer ratio of 1:2.5 displayed in Figure S6(b) exhibited two distinct weight-loss peaks, corresponding to the leakage of core materials and the decomposition of

wall materials, respectively. This further confirms that the particle size distribution and morphological integrity of microcapsules can be significantly improved at an appropriate IPDI/DETA monomer ratio.

Further increasing the dosage of DETA to IPDI/DETA monomer ratios of 1:3 and 1:3.5, the morphology of microcapsules instead deteriorated significantly. Almost no well-formed microcapsules could be observed in Figures S5(c) and S5(d), accompanied by severe agglomeration of wall materials. An excessively high DETA ratio leads to a high concentration of DETA in the aqueous phase. A large number of DETA molecules tend to diffuse into the oil phase from the water phase. The crosslinking reaction between IPDI and DETA will take place in the oil phase rather than confined to the oil-water interface. Massive inhomogeneous polyurea particles are rapidly generated inside the oil phase by the spontaneous bulk polymerization of wall material monomers. Consequently, the final sample is consisted of discrete core material droplets and agglomerated wall materials, rather than microcapsules with core-shell structure. As shown in Figures S6(c) and S6(d), the characteristic peaks corresponding to core materials leakage were inconspicuous. The mass loss of the samples was primarily contributed to the thermal decomposition of wall materials. The TG results were consistent with the SEM observations, further indicating that almost no well-formed microcapsules were generated under such experimental conditions. As concluded, an IPDI/DETA monomer ratio of 1:2.5 was determined as the optimal wall monomer ratio in our study.

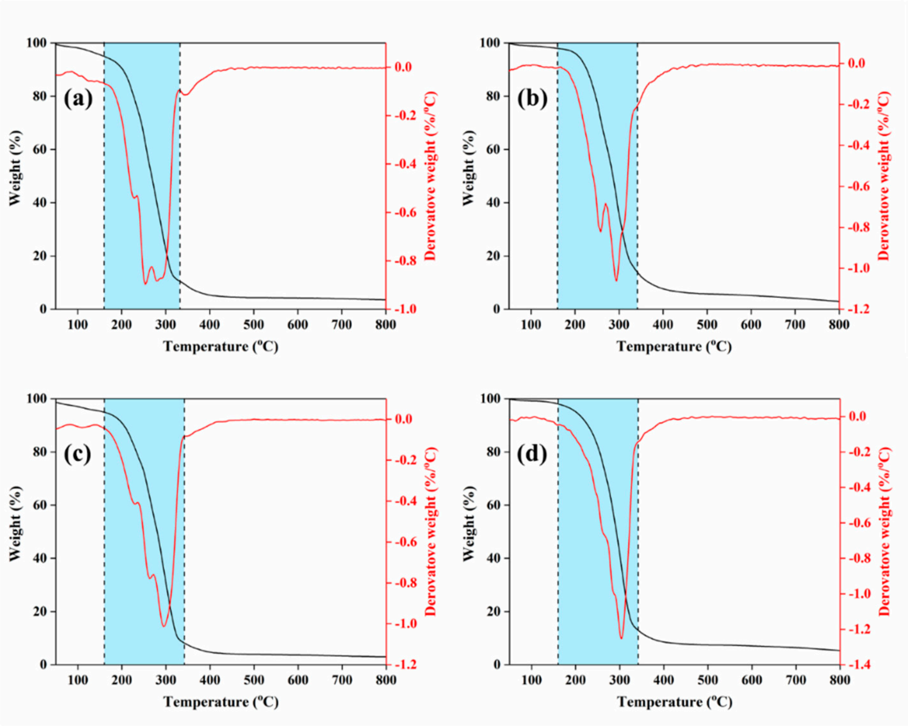

Figure S6. TG and DTG curves of PU@CFO microcapsules prepared at different IPDI/DETA monomer ratios of (a) 1:2, (b) 1:2.5, (c) 1:3, and (d) 1:3.5

S2.4 Effect of CFO loading amount

Loading amount of core materials acts as a vital process parameter affecting the formation performance and core content of microcapsules. Effect of CFO loading amount on the morphology of microcapsules was also investigated at a fixed IPDI/DETA monomer ratio of 1:2.5 under the optimal emulsification conditions. The SEM images and TG curves of the final PU@CFO microcapsules obtained at different CFO loading amounts were demonstrated in Figure S7 and Figure S8, respectively.

When the loading amount of CFO was 2 mL, only a small amount of irregular microcapsules could be observed in the SEM images shown in Figure S7(a). Abundant agglomerated shell particles were formed in the system, indicating poor core content. The relative ratio of core material to shell material is low at low core materials loading amount,

leading to insufficient oil phase and excessive shell monomers in the system. A large number of empty microcapsules without core and discrete polyurea particles were finally generated after the crosslinking reaction. As a result, the yield of microcapsules was relatively low, accompanied by overly thick shells and low core content. Accordingly, the DTG curve in Figure S8(a) exhibited a relatively weak characteristic peak corresponding to the leakage of core materials, and two distinct characteristic peaks corresponding to the decomposition of agglomerated wall materials. As the CFO loading amount increased to 4 mL, the ratio of oil phase to water phase was balanced, providing a stable reaction interface for interfacial polymerization and enabling sufficient crosslinking of shell monomers to form intact and regular core-shell structures. Consequently, the yield of microcapsules and core content were significantly improved simultaneously. As shown in Figure S7(b), most microcapsules exhibited regular spherical morphology with smooth surface and favorable dispersity, without obvious adhesion or agglomeration.

When the CFO loading amount further increased to 6 mL and 8 mL, relatively excess core materials could not be fully encapsulated by limited wall material monomers. As shown in Figure S7(c), the yield of microcapsules decreased obviously, accompanied by numerous irregular agglomerated wall materials particles. Severe self-agglomeration could be observed, especially at CFO loading amount of 8 mL. No intact spherical microcapsules but only massive porous flocculent aggregates could be observed in Figure S7(d), indicating complete destruction of core-shell structure. In general, excessively high core materials dosage leads to insufficient dispersion of numerous core droplets in the continuous aqueous phase. The

frequent collision and coalescence of core droplets significantly reduce the stability of emulsion system. In addition, limited wall material monomers cannot fully encapsulate excess core materials, leading to the formation of microcapsules with excessively thin and even ruptured shell. And the resulting microcapsules tend to self-agglomerate, forming large aggregated clusters. The corresponding DTG curves displayed in Figures S8(c) and S8(d) exhibited obvious advanced weight loss attributed to core material leakage, further indicating the structure destruction of microcapsules at excessively high core materials dosage.

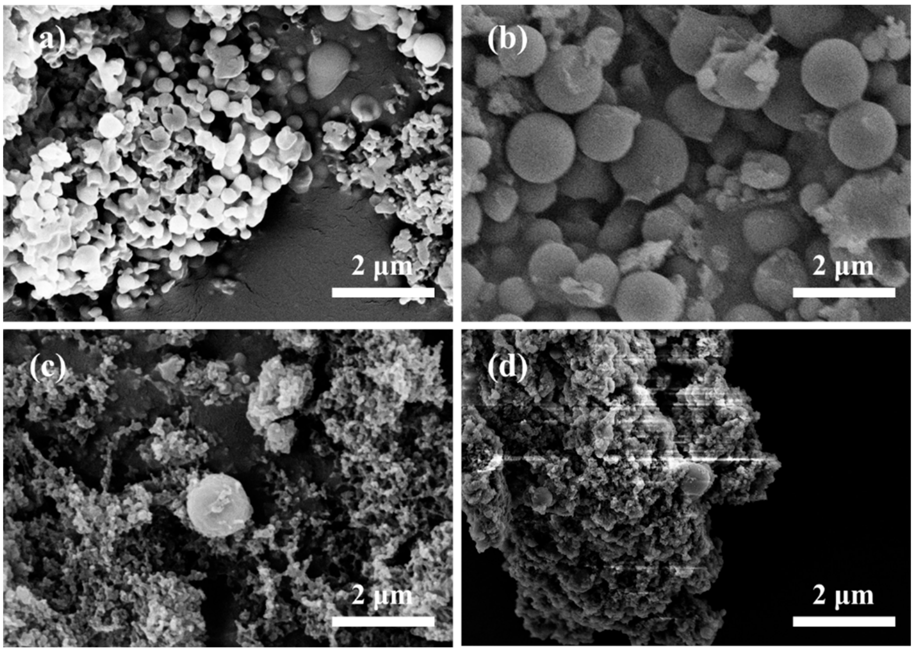

Figure S7. SEM images of PU@CFO microcapsules prepared at different CFO loading amounts of (a) 2 mL, (b) 4 mL, (c) 6 mL, and (d) 8 mL

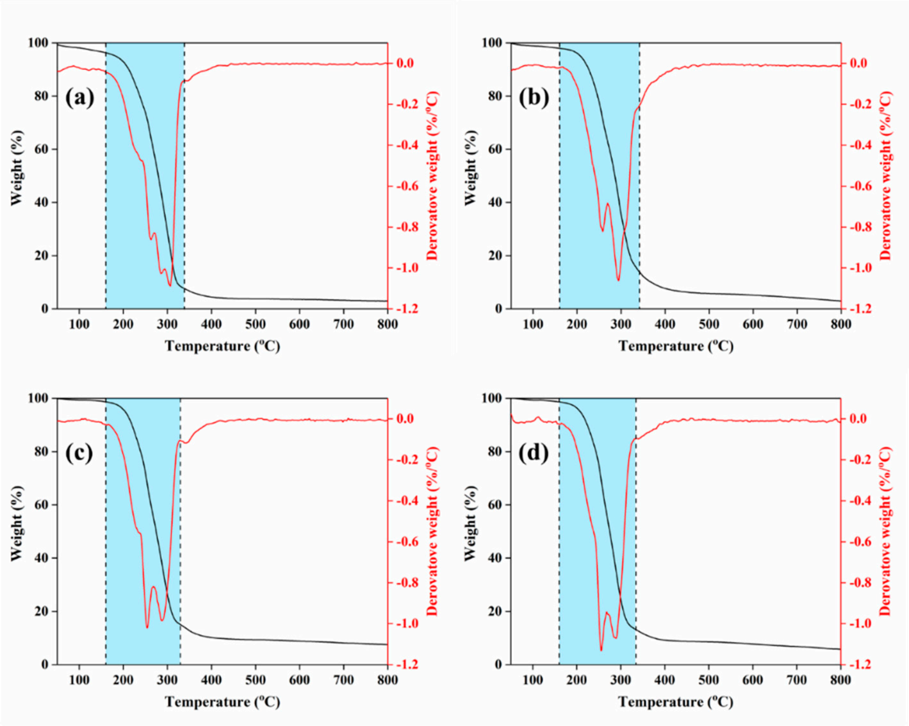

Figure S8. TG and DTG curves of PU@CFO microcapsules prepared at different CFO loading amounts of (a) 2 mL, (b) 4 mL, (c) 6 mL, and (d) 8 mL

Section S3. Core content of PA-MEL@PU@CFO microcapsules

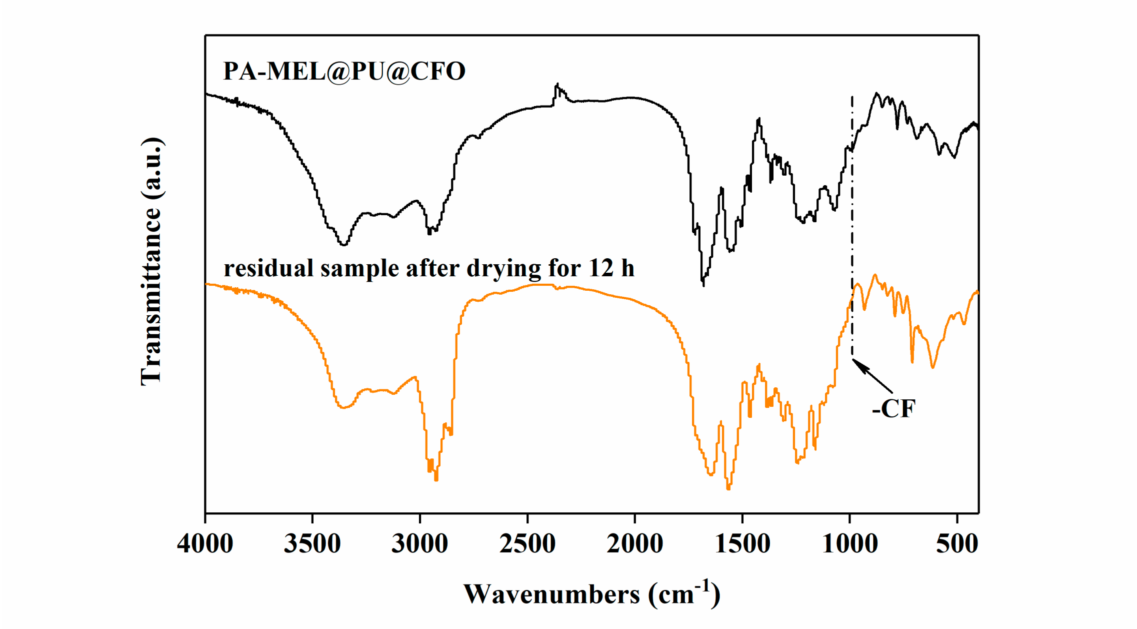

Figure S9. FT-IR spectra of PA-MEL@PU@CFO and the residual sample after grinding and drying at 120 °C for 12 h
